# Asymmetrical‐Dendronized TADF Emitters for Efficient Non‐doped Solution‐Processed OLEDs by Eliminating Degenerate Excited States and Creating Solely Thermal Equilibrium Routes

**DOI:** 10.1002/anie.202115140

**Published:** 2022-01-20

**Authors:** Chensen Li, Alastair K. Harrison, Yuchao Liu, Zhennan Zhao, Cheng Zeng, Fernando B. Dias, Zhongjie Ren, Shouke Yan, Martin R. Bryce

**Affiliations:** ^1^ State Key Laboratory of Chemical Resource Engineering College of Materials Science and Engineering Beijing University of Chemical Technology Beijing 100029 China; ^2^ Chemistry Department Durham University South Road Durham DH1 3LE UK; ^3^ Physics Department Durham University South Road Durham DH1 3LE UK; ^4^ Key Laboratory of Rubber-Plastics Ministry of Education Qingdao University of Science & Technology Qingdao 266042 P.R. China

**Keywords:** Asymmetric, Degenerate excited states, Dendrimer, Non-doped, TADF

## Abstract

The mechanism of thermally activated delayed fluorescence (TADF) in dendrimers is not clear. We report that fully‐conjugated or fully‐nonconjugated structures cause unwanted degenerate excited states due to multiple identical dendrons, which limit their TADF efficiency. We have synthesized asymmetrical “half‐dendronized” and “half‐dendronized‐half‐encapsulated” emitters. By eliminating degenerate excited states, the triplet locally excited state is ≥0.3 eV above the lowest triplet charge‐transfer state, assuring a solely thermal equilibrium route for an effective spin‐flip process. The isolated encapsulating tricarbazole unit can protect the TADF unit, reducing nonradiative decay and enhancing TADF performance. Non‐doped solution‐processed devices reach a high external quantum efficiency (EQE_max_) of 24.0 % (65.9 cd A^−1^, 59.2 lm W^−1^) with CIE coordinates of (0.24, 0.45) with a low efficiency roll‐off and EQEs of 23.6 % and 21.3 % at 100 and 500 cd m^−2^.

## Graphical abstract

Two asymmetric “half‐dendronized” and “half‐dendronized‐half‐encapsulated” TADF emitters eliminate degenerate excited states, assuring a solely thermal equilibrium route for an effective spin‐flip process. AEE properties can minimize the exciton quenching. The isolated encapsulating tricarbazole unit protects the TADF unit, reducing nonradiative decay. Non‐doped OLEDs with a remarkably high EQE_max_ of 24.0 % and low roll‐off are achieved.

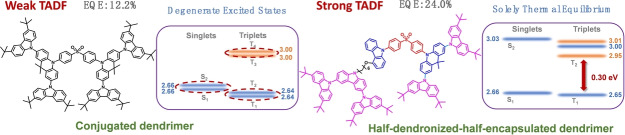

## Introduction

Solution‐processed organic light‐emitting diodes (OLEDs)[^1^, ^2^, ^3^, ^4^, ^5^] offer advantages over vacuum deposition, such as low cost, high processing efficiency, scalability over large areas including flexible substrates, and better control of the doping concentration.[^6^, ^7^] Solution‐processed OLEDs are produced mainly from hosts co‐doped with emitters by physical blending.^[8]^ These doped devices encounter phase separation and inefficient energy transfer, and these defects may limit their electroluminescence (EL) efficiencies and stabilities.^[9]^ Non‐doped devices have a single component in the emission layer with the merits of process simplicity and enhanced stability compared with doped OLEDs.[^10^, ^11^] In particular, this non‐doped technology is relevant to blue emission since the appropriate hosts, requiring large band gaps, are rare.[^12^, ^13^, ^14^, ^15^] There is, therefore, a need for host‐free blue emitters. However, most luminescent materials do not function efficiently in non‐doped OLEDs because of severe concentration quenching and exciton annihilation.[^16^, ^17^] Aggregation‐enhanced emission (AEE)^[18]^ can overcome these drawbacks to achieve efficient solid‐state luminescence. Therefore, synthesis of solution‐processable emitters with AEE properties is a promising strategy.

Three generations of efficient emitters have been developed.[^19^, ^20^, ^21^] The first generation fluorescent emitters are limited to 25 % internal quantum efficiency (IQE)^[22]^ while the second generation phosphorescent emitters suffer from the use of expensive noble metals (e.g., Ir,^[23]^ Pt^[24]^ or Au^[25]^). The third generation of thermally activated delayed fluorescence (TADF) materials are especially promising due to the theoretical 100 % exciton harvesting of both singlet (S_1_) and triplet (T_1_) excitons.[^26^, ^27^] In the TADF process, triplet excited states are converted to singlet states through reverse intersystem crossing (RISC). A fast RISC rate enables this process to outcompete the non‐radiative decay rate,^[28]^ and thereby to achieve a high total fluorescence yield, combining prompt and delayed fluorescence. TADF molecules typically comprise electronically decoupled electron donor (D) and electron acceptor (A) subunits. This topology leads to the separation of the highest occupied and lowest unoccupied molecular orbitals (HOMO and LUMO, respectively) on the emitter, and to the formation of intramolecular charge transfer (CT) states with a small thermally accessible energy gap (Δ*E*_ST_) between the S and T states toward a small electron exchange energy.^[27]^ The different energy alignment between adjacent excited states can also influence the TADF rate.^[29]^ A small triplet–triplet energy gap (Δ*E*_T1‐T2_) could lead to unnecessary thermal equilibrium between adjacent singlet and triplet energy levels, eventuating in undesired nonradiative processes, which are unfavorable to the RISC process.^[30]^ Alternatively, a large Δ*E*_T1‐T2_ could ensure solely thermal equilibrium processes without interference from high‐lying excited states, which is beneficial to a fast RISC rate. Therefore, avoiding multiple close thermal equilibrium processes to enlarge the band gap between adjacent excited states is a significant factor to design efficient TADF emitters.

Dendronized emitters benefit from good solubility, high‐quality films and diversity of molecular designs. For fluorescent dendrimers, the EQEs of OLEDs are limited to 5 % due to effective intermolecular interactions and exciton quenching promoted by aggregation.^[31]^ However, for phosphorescent dendrimers, the EQE of non‐doped devices based on metallodendrimers achieved 21.2 %,^[32]^ which is comparable to the most efficient TADF devices (20.4 %).^[33]^ Therefore, there is potential to enhance the efficiency of TADF dendrimer devices. Generally, TADF dendrimers are derived from TADF small molecules by integrating dendrons to extend the molecular size. However, most dendrimers do not retain the high photoluminescent (PL) or electroluminescent (EL) efficiencies of the original small molecules. For the conjugated linkages strategy,^[34]^ excessive dendrons can cause the HOMO to localize predominantly over the outer dendrons, consequently weakening the CT and TADF properties; besides, this kind of dendrimer is likely to have a significant red tail in the EL attributed to excimer formation.^[35]^ For a non‐conjugated linkage strategy, more flexible chains and outer dendrons lead to longer radiative lifetimes, which might increase unwanted efficiency roll‐off at high luminance.^[33]^ The inherent factors that constrain the efficiency of these symmetric dendrimers are still not clear. A new dendronized emitter design strategy is needed to open a new direction for TADF macromolecules.

**Figure 1:**
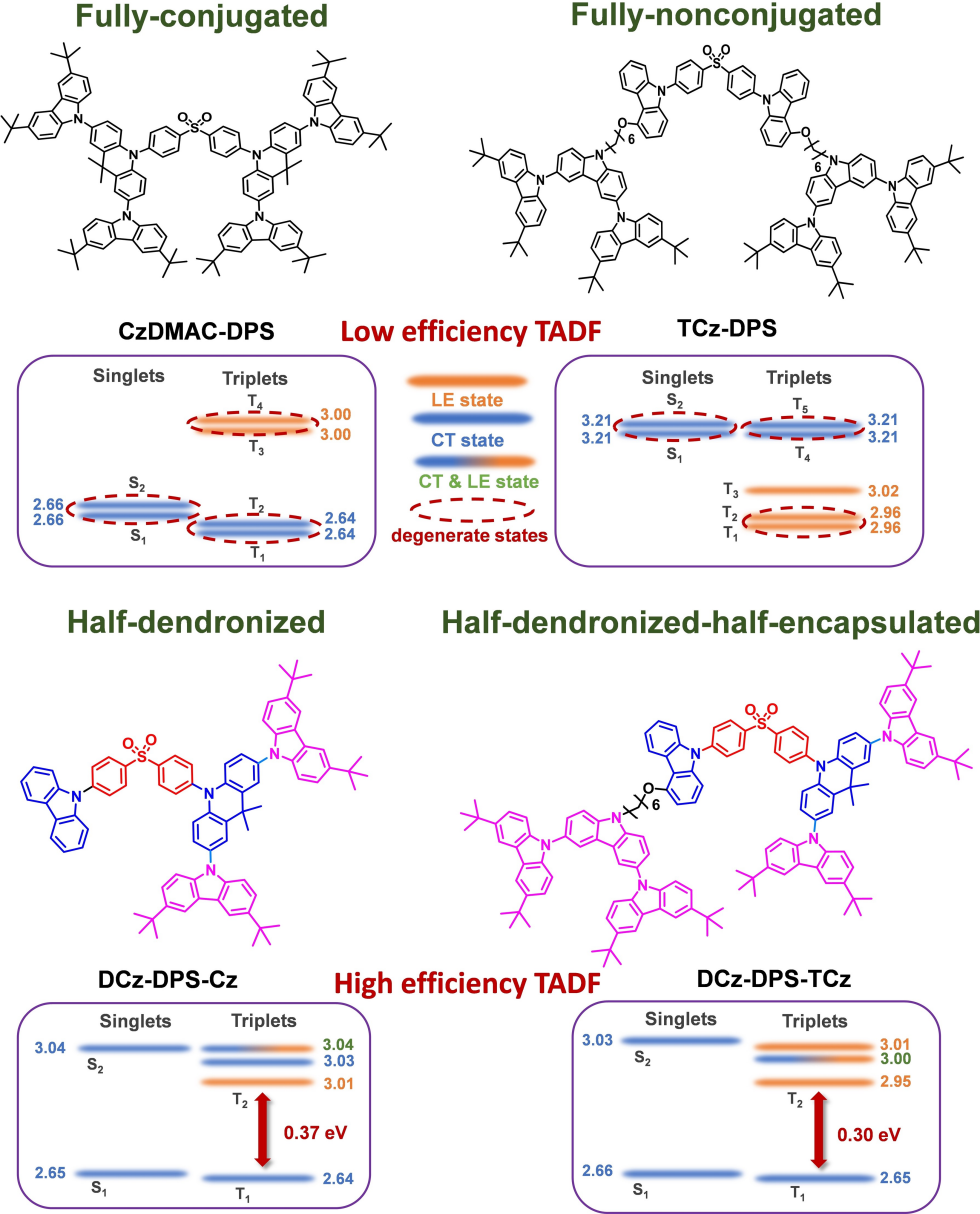
Molecular structures and excited states of fully‐conjugated and fully‐nonconjugated symmetrical dendrimers **CzDMAC‐DPS** and **TCz‐DPS**, and asymmetrical half‐dendronized **DCz‐DPS‐Cz** and **DCz‐DPS‐TCz**.

Diphenylsulfone (DPS) as a TADF acceptor has been widely used in constructing dendrimers, but no highly efficient TADF DPS dendrimer has been reported.^[29]^ For example, conjugated and nonconjugated dendrimers **CzDMAC‐DPS** and **tbCz‐SO** based on DPS exhibit low OLED performance with EQE_max_ values of 12.2 %^[35]^ and 2.6 %,^[36]^ respectively. To understand the limiting factors behind these dendrimers, in the present work two symmetric dendrimers, **CzDMAC‐DPS** and modified **tbCz‐SO** with tricarbazole dendrons **TCz‐DPS**, and two asymmetric dendrimers, **DCz‐DPS‐Cz** and **DCz‐DPS‐TCz**, have been investigated by theoretical calculations. Two new types of TADF compounds have been synthesized; their structures are shown in Figure 1. Based on an asymmetric TADF small molecule, 10‐(4‐((4‐(9*H*‐carbazol‐9‐yl)phenyl)sulfonyl)‐phenyl)‐9,9‐dimethyl‐9,10‐dihydroacridine (**CzAcSF**),^[37]^ the dendron sub‐structure “half‐dendronized” **DCz‐DPS‐Cz** has been synthesized. We reasoned that the substituents feature a gradient of the HOMO level resulting in a favorable HOMO‐LUMO overlap between peripheral electron‐rich units and an electron‐accepting core to realize effective TADF.^[38]^ These emitters exhibit AEE properties,^[39]^ which could minimize the exciton quenching in aggregated states, and consequently reduce efficiency roll‐off at high luminance in OLEDs. Importantly, the excited states of the asymmetric dendronized emitters have large Δ*E*_S1‐S2_ and Δ*E*_T1‐T2_ values, which could reduce the thermal equilibrium process between adjacent singlet and triplet energy levels, resulting in decreased nonradiative processes and improved TADF performance. **DCz‐DPS‐TCz** has been obtained by non‐conjugated functionalization of **DCz‐DPS‐Cz** with a branched tricarbazole unit to construct a “half‐dendronized‐half‐encapsulated” structure. Their detailed photophysical properties and non‐doped solution‐processed OLEDs are reported.

## Results and Discussion

### Molecular Design and Synthesis

The syntheses of **DCz‐DPS‐Cz** and **DCz‐DPS‐TCz** are described in the Supporting Information (Schemes S1 and S2). The structures of these molecules, and of the intermediates, were established by ^1^H‐ and ^13^C NMR spectroscopy, mass spectrometry, and elemental analysis. **DCz‐DPS‐Cz** and **DCz‐DPS‐TCz** have good solubility in chloroform, toluene, dichloromethane, chlorobenzene, and tetrahydrofuran. Their thermal decomposition temperatures (*T*_d_) with 5 % weight loss are 422 and 445 °C by TGA, indicating good thermal stability (Supporting Information, Figure S21, Table S1).

**Figure 2:**
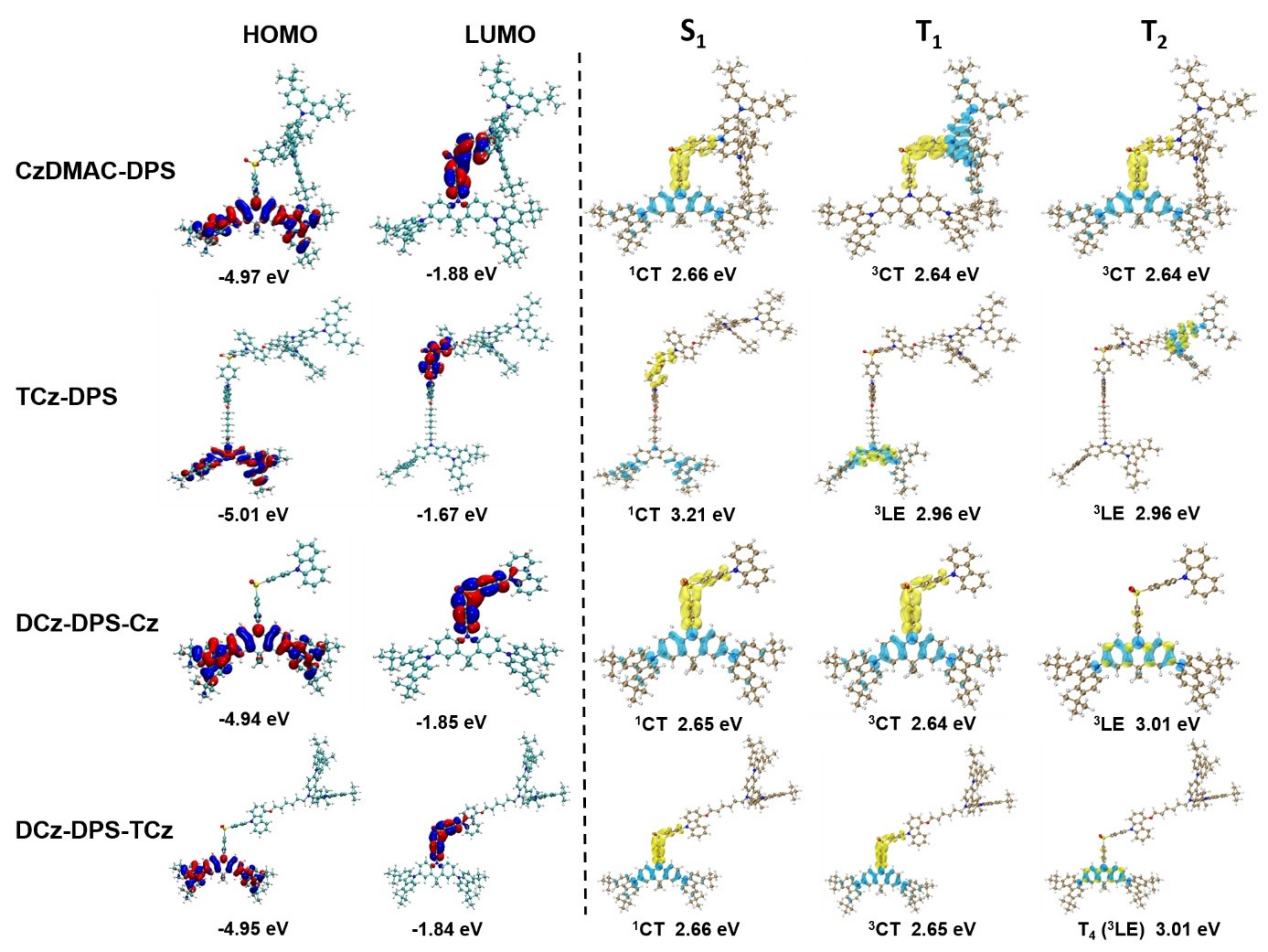
Frontier orbital distributions and NTO analysis of **CzDMAC‐DPS**, **TCz‐DPS**, **DCz‐DPS‐Cz**, and **DCz‐DPS‐TCz**.

The molecular simulation of these emitters by density functional theory (DFT) shows the minimum energy molecular conformations and the HOMO and LUMO distributions (Figure 2; Figure S22). For **CzDMAC‐DPS, DCz‐DPS‐Cz**, and **DCz‐DPS‐TCz**, the conjugation of the 3,6‐di‐*tert*‐butylcarbazole units with acridan enhances the electron donating ability, and therefore the HOMO distribution is mainly on these three subunits, while the LUMO is mainly distributed on the diphenylsulfone acceptor. Due to the half‐conjugated structure, **DCz‐DPS‐Cz** and **DCz‐DPS‐TCz** show less overlap between HOMO and LUMO. For nonconjugated **TCz‐DPS**, the HOMO and LUMO are separately distributed on the isolated tricarbazoles and DPS acceptor. The long distance between HOMO and LUMO causes a very small overlap integral of frontier orbitals, which results in a small radiative transition rate. The overlap integrals in **DCz‐DPS‐Cz** and **DCz‐DPS‐TCz** are only 0.085 and 0.081, respectively, which is obviously lower than that of **CzDMAC‐DPS** (0.096) and higher than **TCz‐DPS** (0.00062). This means that the half‐dendronized strategy is efficient to optimize the overlap between the HOMO and LUMO.

Time dependent DFT (TD‐DFT) analysis using B3LYP/6‐31G(d) by Gaussian 09 and natural transition orbital (NTO) analysis were performed to examine the nature of the excited states by Multiwfn.^[40]^ Notable features of the symmetric dendrimers, **CzDMAC‐DPS** and **TCz‐DPS**, and the asymmetric dendronized emitters, **DCz‐DPS‐Cz** and **DCz‐DPS‐TCz**, are: (i) for **CzDMAC‐DPS**, **DCz‐DPS‐Cz**, and **DCz‐DPS‐TCz**, ^1^CT and ^3^CT states are separately distributed on the DMAC donor and DPS acceptor, leading to a small Δ*E*_ST_ of 0.02, 0.007, and 0.006 eV, respectively; for **TCz‐DPS**, the hole and electron of ^1^CT states are separately distributed on the isolated tricarbazoles and DPS acceptor, and the lowest ^3^LE states are localized on the isolated tricarbazoles, resulting in a larger Δ*E*_ST_ of 0.25 eV, which is unfavorable for an efficient TADF process. (ii) For symmetric dendrimers **CzDMAC‐DPS** and **TCz‐DPS**, S_2_ and T_2_ are almost energetically equivalent to the lowest ^1^CT and ^3^CT states and lowest ^1^CT and ^3^LE states, respectively. These degenerate states are ubiquitous in conventional TADF dendrimers since the multiple repeated dendron units can cause near‐identical charge transfer from dendron donors to acceptors, which can lead to unnecessary thermal equilibrium between adjacent singlet and triplet energy levels, resulting in undesired nonradiative processes with low TADF efficiency. As reported previously,^[29]^ the rate constants of TADF (*k*_TADF_) can be estimated using Equations 1, 2 and 3 in the case of Δ*E*_ST_<0.3 eV and *T*=300 K:

(1) $$k{}_{\mathrm{TADF}}\approx k{}_{F}\;K/\left(1+K{}^{'}\right)$$

(2) $$K=1/3\;\exp(-\Delta E{}_{\mathrm{ST}}/k{}_{B}\;T)$$

(3) $$K{}^{'}=\exp\left(-\Delta E{}_{\mathrm{TT}}/k{}_{B}\;T\right)$$

Where *K* and *K*′ are Boltzmann partition coefficients between the singlet and triplet states (*K*) and the two triplet states (*K*′); *k*_F_ is the rate constant of fluorescence; *k*_B_ is the Boltzmann constant, and Δ*E*_TT_ is the energy difference between the two triplet states. Therefore, a relatively large *K* and a small *K*′ value are favorable to promote the TADF process, which is a consequence of a small Δ*E*_ST_ as well as a large Δ*E*_TT_. The calculated *K* and *K*′ values of **CzDMAC‐DPS** were 0.297 and 0.679; the values of **TCz‐DPS** were 2.19×10^−5^ and 0.962 [Eqs. (2) and (3)]. Obviously, these values are unsatisfactory to achieve a highly efficient TADF performance.

However, for the asymmetric dendronized emitters **DCz‐DPS‐Cz** and **DCz‐DPS‐TCz**, the S_2_ and T_2_ states are higher in energy than the corresponding S_1_ and T_1_ states by ≥0.3 eV. The Boltzmann partition relationship indicates that the thermal equilibrium states between two energy levels with a gap as large as 0.3 eV could be very small, followed by an instant IC process once excitons are generated on high‐lying excited states (Figure 1). The calculated *K* values were around 0.254 and 0.264, and *K*′ values were 6.33×10^−7^ and 1.02×10^−5^ at 300 K, respectively, meaning Equation (1) can be simplified to *k*_TADF_=*k*_F_ *K*. In this case, a fast TADF process could occur by eliminating degenerate excited states and creating the solely thermal equilibrium process in the asymmetrical dendronized emitters.

Furthermore, the SOC matrix element (SOCME) values of ⟨*S_1_|Ĥ_SOC_|T_1_* ⟩ are 0.028 and 0.030 cm^−1^ for **DCz‐DPS‐Cz** and **DCz‐DPS‐TCz**, respectively, which means the values are small between ^3^CT and ^1^CT; therefore, RISC is less likely to occur through this route in light of El‐Sayed's rule. For **DCz‐DPS‐Cz**, the higher T_2_ (^3^LE) is localized in the DMAC, and T_4_ (^3^CT and ^3^LE) is distributed in the partially overlapped carbazole and DPS (Figure S22). The SOCME values ⟨*S_1_|Ĥ_SOC_|T_2_* ⟩ and ⟨*S_1_|Ĥ_SOC_|T_4_* ⟩ are 0.85 and 0.35 cm^−1^ with Δ*E*_ST_ values of 0.36 and 0.39 eV, respectively. For **DCz‐DPS‐TCz**, the hole and electron of T_2_(^3^LE) are localized in the isolated tricarbazole, which is positioned too far to mediate the spin flip from the triplet state to the singlet state. The T_3_ (^3^CT and ^3^LE) is distributed in partially overlapped carbazole and DPS, and the T_4_ (^3^LE) is mainly localized in DMAC. The SOCME values ⟨*S_1_|Ĥ_SOC_|T_3_* ⟩ and ⟨*S_1_|Ĥ_SOC_|T_4_* ⟩ are 0.36 and 0.88 cm^−1^, with Δ*E*_ST_ values of 0.34 and 0.35 eV, respectively. Due to the valid RISC channels with both small Δ*E*_ST_ (≤0.37 eV) and high SOC values (≥0.3 cm^−1^),^[41]^ the number of RISC channels of **DCz‐DPS‐Cz** and **DCz‐DPS‐TCz** were determined to be 1 (T_2_) and 2 (T_3_ and T_4_), respectively. Therefore, the reasonable mechanism in first‐order approximation is that the RISC process from triplet to ^1^CT would be higher ordered single/multiple channel spin‐flip processes, which involves the second‐order nonadiabatic vibronic coupling from T_1_ to T_*n*,*n*>1_, followed by the spin‐flipping process from T_*n*,*n*>1_ to S_1_.[^42^, ^43^]

**Figure 3:**
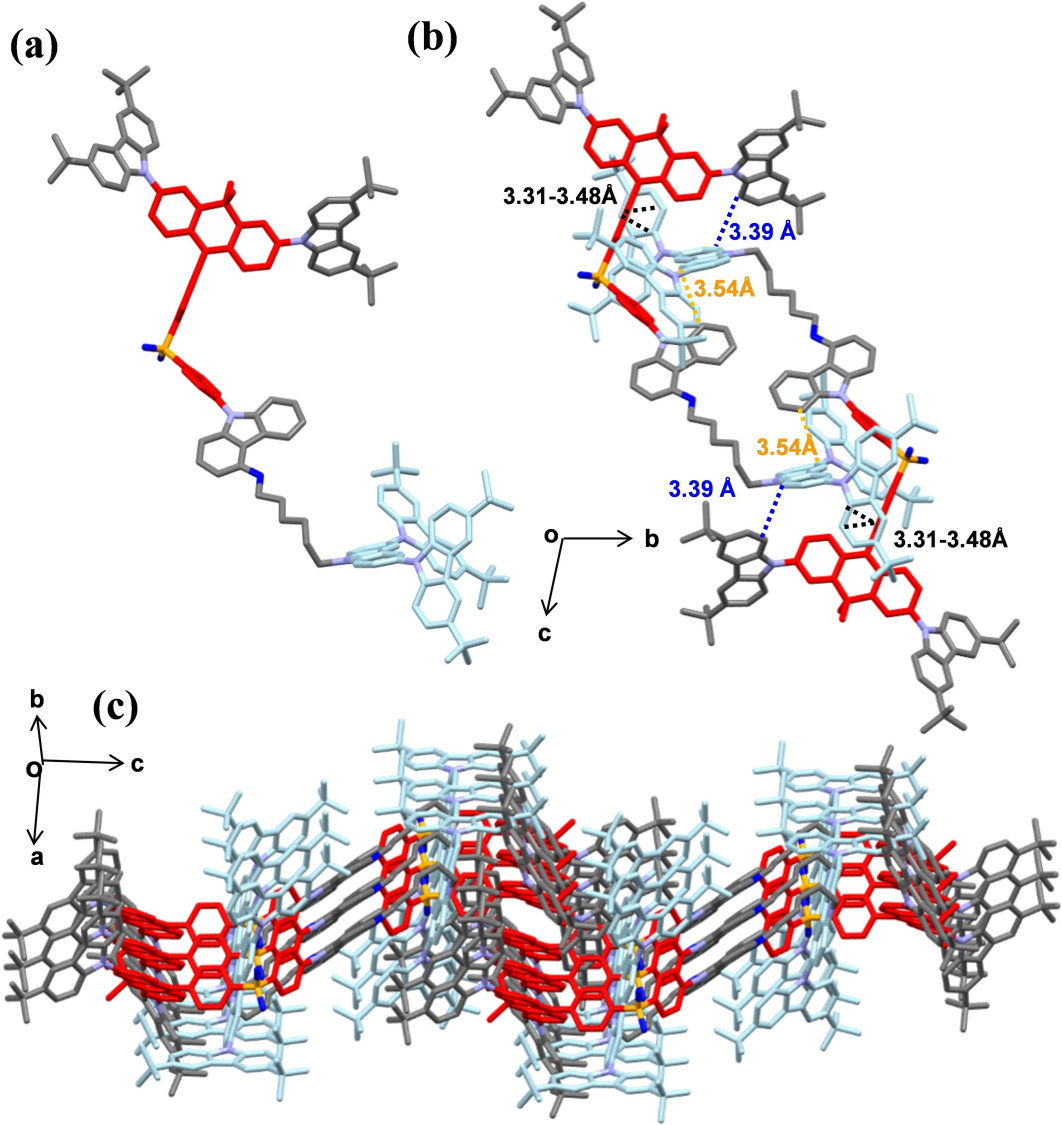
Single‐crystal structure and packing diagrams of **DCz‐DPS‐TCz**.

Single crystals of **DCz‐DPS‐TCz** were obtained by the slow solvent vapor diffusion method (dichloromethane/*n*‐hexane). Upon standing at ambient temperature, single crystals were observed, indicating the thermodynamically stable crystalline state (Figure 3).^[44]^ The molecule has a stretched conformation, which is very similar to the simulated results (Figure 2). **DCz‐DPS‐TCz** forms bimolecular stacks with π‐π interactions. Each DPS acceptor is encapsulated by a tricarbazole unit of an adjacent molecule with a mean plane distance of 3.31–3.48 Å (Figure 3b). The mean distances between the isolated tricarbazole with dendronized carbazole and donor carbazole are 3.39 and 3.54 Å, respectively. The TADF units are surrounded by carbazole groups (Figure 3c). The intermolecular distance between two adjacent DPS units is 11.58 Å. These results suggest the half‐dendronized‐half‐encapsulated strategy can effectively protect TADF cores from intermolecular exciton quenching. The crystal shows red‐shifted absorption and blue‐shifted PL compared with the neat film (Figure S23) indicating that the crystal provides a rigid environment and restrains the vibration and rotation of molecules, leading to a smaller Stokes shift.

### Photophysical Properties

**Figure 4:**
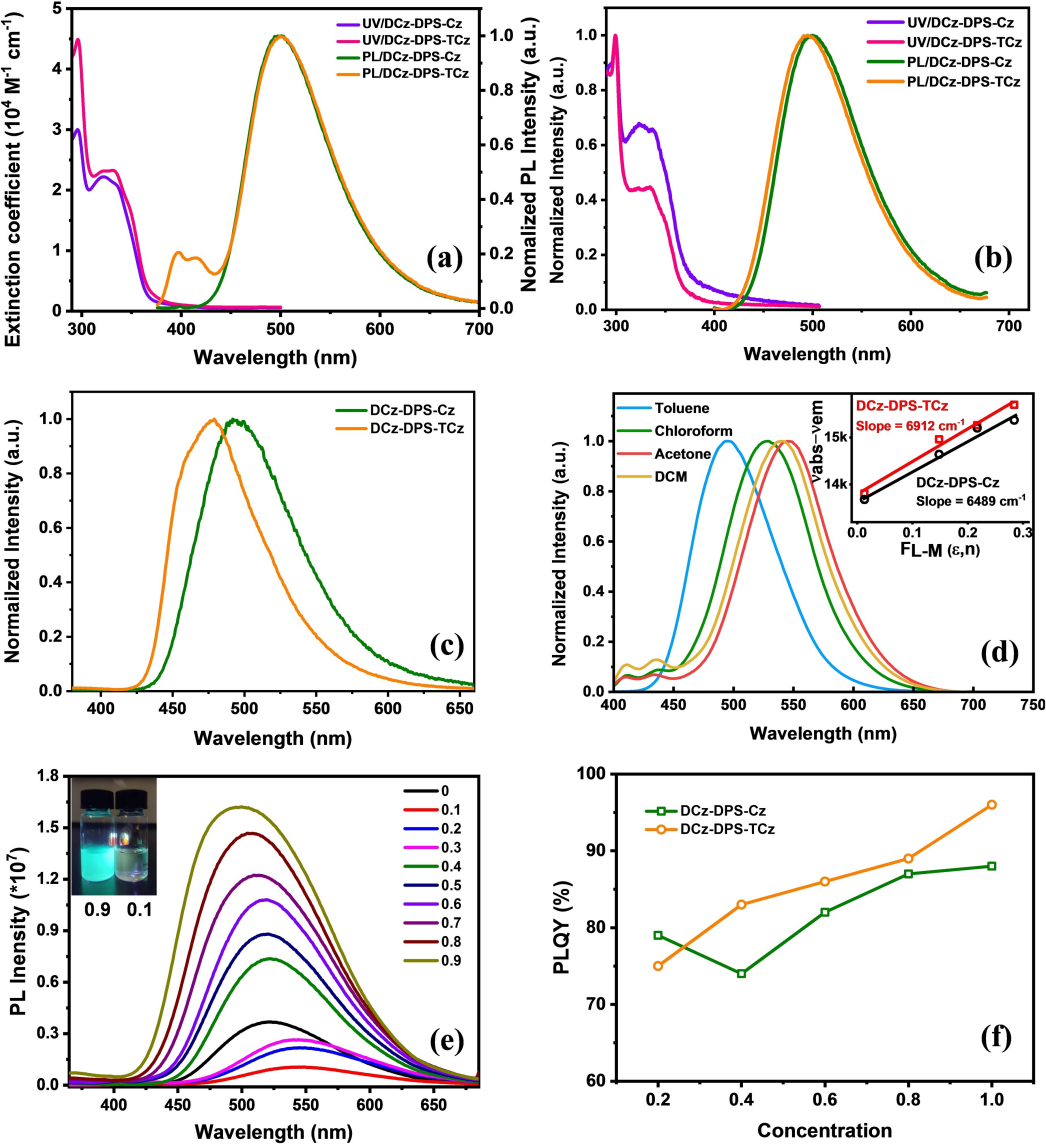
UV/Vis absorption and PL spectra in a) toluene solution and b) neat films of the two compounds. c) Phosphorescence spectra collected at 77 K of neat films of **DCz‐DPS‐Cz** and **DCz‐DPS‐TCz**. d) PL spectra in toluene, chloroform, acetone, and dichloromethane (DCM) solutions of **DCz‐DPS‐Cz** and the corresponding Lippert–Mataga plot of the two emitters (inset). e) PL spectra of DCz‐DPS‐Cz in water/THF mixtures with different fractions of water; the inset is fluorescent images of 0.9 and 0.1 water/THF solution mixtures under UV light irradiation. f) Plots of PLQYs in different ratios of the two emitters in doped PMMA films. All the PL spectra were excited at 355 nm.

Figure 4 shows the UV/Vis absorption and fluorescence spectra of **DCz‐DPS‐Cz** and **DCz‐DPS‐TCz**. In toluene solution (Figure 4a) the bands around 300 nm are mainly attributed to the π‐π* transition of carbazole units,^[45]^ and the bands at 320–370 nm are mainly attributed to tricarbazole, or the electron donor/acceptor units in both compounds.[^45^, ^46^, ^47^] **DCz‐DPS‐TCz** shows the stronger absorption and higher molar absorption coefficient, which is consistent with the oscillator strengths of S_1_ of **DCz‐DPS‐Cz** (0.029) and **DCz‐DPS‐TCz** (0.039),^[48]^ which means the transition from S_1_ to S_0_ of **DCz‐DPS‐TCz** is more active than that of **DCz‐DPS‐Cz**. The fluorescence spectra of dilute toluene solutions of both emitters show peaks at ≈498–500 nm with charge transfer characteristics (Figure 4a). In addition, in toluene **DCz‐DPS‐TCz** shows a blue‐shifted fluorescence band assigned to the tricarbazole unit at ≈380–430 nm (Figure 4a). This unit is separated from the TADF segment by the non‐conjugated hexyloxy spacer, and the tricarbazole appears not to be involved in intramolecular charge transfer in dilute solution. Their fluorescence spectra in neat films show similar profiles, although the tricarbazole emission at ≈380–430 nm of **DCz‐DPS‐TCz** is not observed. This could indicate stronger charge transfer from tricarbazole to the TADF unit in the aggregated state, or quenching of the tricarbazole emission due to energy transfer.

The phosphorescence spectra of **DCz‐DPS‐Cz** and **DCz‐DPS‐tCz** of neat films at 77 K are shown in Figure 4c. The Δ*E*_ST_ values are ≈0.03 eV, calculated from the onset energy of the main CT‐type spectra, which is much lower than that for **CzAcSF** (0.07 eV),^[37]^ indicating that the half‐dendronized strategy is effective to decrease Δ*E*_ST_. These very small Δ*E*_ST_ values indicate that the emitters should readily undergo RISC and be TADF‐active. The two compounds show positive solvatochromism of their emission spectra in toluene, chloroform, acetone, and dichloromethane (Figure 4d, Table 1; Figure S23). Apart from the red‐shifted CT‐type emission in **DCz‐DPS‐Cz**, a small emission band at 400–450 nm is seen in polar solvent, ascribed to LE‐type emissions from acridan with carbazole dendron units. For **DCz‐DPS‐TCz**, the blue‐shifted emission at *λ*_max_ ca. 400 nm is dominated by the tricarbazole units. This emission is less affected by increasing polarity due to its LE character. The red‐shifted emission, at *λ*_max_ ca. 520 nm, is highly quenched with increasing solvent polarity due to its CT character. Therefore, the relative intensity of the two emission bands varies with increasing polarity. The Lippert–Mataga plots (ν_abs_‐ν_em_ against polarity of solvent) exhibit a slope of ≈6489 cm^−1^ and 6912 cm^−1^ for **DCz‐DPS‐Cz** and **DCz‐DPS‐TCz**, respectively, indicating that the latter shows enhanced CT character.^[49]^

**Table 1:** Summary of photophysical data for **DCz‐DPS‐Cz** and **DCz‐DPS‐TCz**.

Emitter	*λ*_em_ [nm]^[a]^	*E*_g_ [eV]^[b]^	HOMO [eV]^[c]^	LUMO [eV]^[d]^	τ_PF_ */ϕ*_PF_ [ns/%]^[e]^	τ_DF_ */ϕ*_DF_ [μs/%]^[e]^	*Φ*_PL_ [%]^[f]^	S_1_/T_1_ [eV]^[g]^	Δ*E*_ST_ [eV]^[h]^	*k*_RISC_ [10^6^ s^−1^]^[i]^	$k_{nr}^{T}$ [10^5^ s^−1^]^[j]^
**DCz‐DPS‐Cz**	498/494	3.37	−5.20	−1.83	34/21	1.51/79	85/88	2.97/3.00	0.03	2.6	2.00
**DCz‐DPS‐TCz**	500/500	3.37	−5.22	−1.85	22/17	1.43/83	90/96	2.97/3.00	0.03	3.0	1.35

[a] The peak values of PL spectra measured in dilute toluene and neat films at room temperature, respectively. [b] Optical energy gap (*E*_g_) deduced from the absorption onset in toluene. [c] Calculated according to the equation *E*_HOMO_=−(*E*_(onset, o*x* vs Fc+/Fc)_+4.8) by CV. [d] Calculated according to the equation LUMO=HOMO+*E*_g_. [e] The lifetime and ratio of prompt and delayed fluorescence component. [f] Absolute PL quantum yield in toluene solution and in thin films, respectively, determined by a calibrated integrating sphere in degassed conditions; error ±2 %. [g] Singlet and triplet energies were determined from the onset wavelength of fluorescence at RT and phosphorescence at 77 K in neat films. [h] Singlet–triplet energy gap and Δ*E*_ST_=S_1_‐T_1_. [i] The rate constant of reverse intersystem crossing calculated from *k*_RISC_=Φ_PL_/(*τ*_DF_×(1−Φ_DF_)). [j] The nonradiative decay rate of triplet exciton calculated from $k_{nr}^{T}$ =(1−Φ_DF_)/*τ*_DF_.

The very weak emission of **DCz‐DPS‐Cz** and **DCz‐DPS‐TCz** in pure THF solution increases steadily on increasing the content of water, indicating AEE, possibly due to aggregation of the TADF core (Figure 4e; Figure S24). The twisted conformation of TADF units^[18]^ will favor loose packing with weak molecular interactions, and thus rotation and vibration will occur easily in dilute solution. In contrast, in the aggregated state, intramolecular motions are restricted, and thus the nonradiative pathways of the excited state are blocked.^[18]^ Similar optical properties can also be observed in doped poly(methyl methacrylate) (PMMA) films (Figure 4f). The PLQYs of the two emitters increase from 79 % and 75 % with 80 % PMMA (0.2 concentration) to 88 % and 96 % without PMMA (1.0 concentration).

Solution electrochemistry was investigated by cyclic voltammetry (CV; Figure S25). **DCz‐DPS‐Cz** exhibits two quasi‐reversible oxidation processes, assigned to the oxidation of DMAC and carbazole dendrons, respectively. **DCz‐DPS‐TCz** exhibits three quasi‐reversible oxidation processes; the additional oxidation process for **DCz‐DPS‐TCz** can be assigned to the tricarbazole dendron, which is consistent with the DFT simulation of the HOMO‐1 orbital. A slight increase in oxidation potential is observed for **DCz‐DPS‐TCz** (0.42 eV) compared to **DCz‐DPS‐Cz** (0.40 eV). The HOMO levels of **DCz‐DPS‐Cz** and **DCz‐DPS‐TCz** can be calculated as −5.20 and −5.22 eV, respectively, which is higher than that of **CzAcSF** (−5.89 eV).^[37]^ Since one side of the acceptor DPS of the two emitters is the donor acridan with dicarbazole dendrons, the gradient of the HOMO levels from acridan to dicarbazole dendrons is expected.

**Figure 5:**
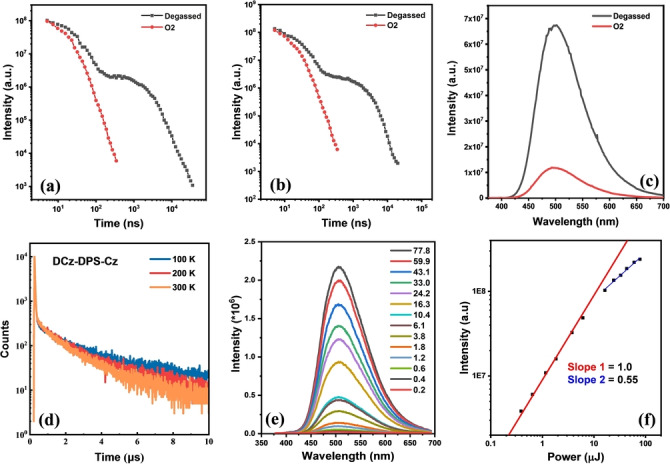
PL decays in degassed and oxygenated toluene solution of a) **DCz‐DPS‐Cz** and b) **DCz‐DPS‐TCz**. c) PL spectra in degassed and oxygenated toluene solution of **DCz‐DPS‐Cz**. d) PL decays in neat films of **DCz‐DPS‐Cz**. e,f) Dependence of DF intensity with excitation power and PL spectra of **DCz‐DPS‐Cz** (0.2–77.8 μJ) in neat films. These PL transient spectra were collected at 500 nm with 355 nm excitation.

Figure 5a,b show the PL decays in toluene with and without oxygen. These data confirm TADF performance. In the absence of oxygen, the emitters show double exponential decay curves of the prompt fluorescence (PF) in the nanosecond regime and the delayed fluorescence (DF) in the microsecond regime, whereas both compounds show only a single exponential decay in the presence of oxygen. The DF : PF ratio was determined from integration of the steady‐state spectra obtained in degassed and aerated conditions (Figure 5c; Figure S26a). Oxygen mainly quenches triplets that are involved in DF. The luminescence of both **DCz‐DPS‐Cz** and **DCz‐DPS‐TCz** significantly increased upon the removal of oxygen with no observable change in the profile of the spectra and no significant change in *λ*_max_, suggesting little influence from local excited states and providing more evidence that CT is responsible for the emission.

The DF : PF ratio for **DCz‐DPS‐TCz** (4.86) is higher than for **DCz‐DPS‐Cz** (3.82). A plausible explanation is that the flexible alkyl linker of **DCz‐DPS‐TCz** enables the tricarbazole unit to encapsulate the central TADF unit and reduce the quenching of the triplet state by oxygen, resulting in stronger TADF. Steady‐state emission in oxygen and non‐oxygenated neat films (Figure S27) revealed no significant difference in emission intensity in these conditions, suggesting there is low oxygen permeability in neat films, probably owing to a protective effect of peripheral carbazole units for the TADF core. The delayed fluorescence (DF) lifetimes of **DCz‐DPS‐Cz** and **DCz‐DPS‐TCz** in neat films decreased from 2.30 and 1.90 to 1.51 μs, and from 1.89 and 1.61 to 1.43 μs, respectively, with increasing temperature from 100 to 300 K (Figure 5d; Figure S26b). The DF lifetimes show obvious temperature dependence, suggesting an effective RISC process and excellent TADF properties. The two emitters show a significantly shorter DF lifetime compared with **CzAcSF** (5.6 μs),^[37]^ which means our strategy decreases both DF lifetime and Δ*E*_ST_ and is thus conducive to the RISC process. The PLQYs of **DCz‐DPS‐Cz** and **DCz‐DPS‐TCz** achieve 85 % and 90 %, respectively, in degassed toluene. Indeed, the RISC rates (*k*_RISC_) were 2.6×10^6^ and 3.0×10^6^ s^−1^, for **DCz‐DPS‐Cz** and **DCz‐DPS‐TCz**, respectively (Table 1). **DCz‐DPS‐TCz** also exhibits a smaller nonradiative decay rate of triplet excitons ($k_{nr}^{T})$ than **DCz‐DPS‐Cz** (1.35×10^5^ vs. 2.00×10^5^ s^−1^). Therefore, **DCz‐DPS‐TCz** shows a higher *k*_RISC_ rate, lower $k_{nr}^{T}$ rate, and enhanced TADF properties owing to the encapsulated tricarbazole preventing exciton quenching. The integral of the DF of the two emitters in neat films (150 ns delay time and integrated over 200 ns) shows a linear dependence (gradient 1) with excitation power, confirming that the origin of DF is a monomolecular process; that is, TADF (Figure 5e,f; Figure S26c,d). This data excludes the possibility of a bimolecular process such as triplet–triplet annihilation (TTA).^[50]^ When the power was increased to ≥10 μJ, the slope decreased to ≈0.5, indicating some degree of quenching (possibly due to singlet–singlet or triplet–triplet annihilation).

### OLED Performance

**Figure 6:**
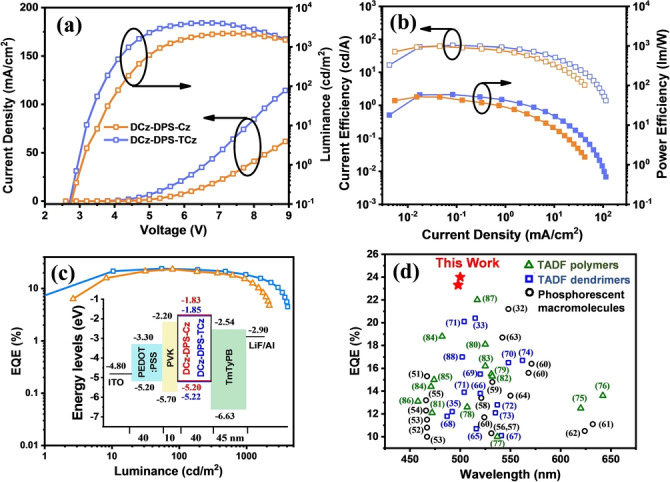
a) Current density‐voltage‐luminance curves. b) Current efficiency‐luminance‐power efficiency curves. c) External quantum efficiency versus luminance curves of the devices. Inset: energy‐level diagrams and structures of OLED devices. d) The current state‐of‐the‐art EQE for phosphorescent and TADF macromolecular (dendrimer and polymer) non‐doped OLEDs, versus *λ*max^EL^.

Non‐doped OLEDs were fabricated by solution processing with a configuration of ITO/poly(3,4‐ethylenedioxythiophene): poly(styrenesulfonate) (PEDOT : PSS) (40 nm)/poly(*N‐*vinylcarbazole) (PVK) (10 nm)/ **DCz‐DPS‐Cz** (Device A) or **DCz‐ DPS‐TCz** (Device B) (EML) (40 nm)/1,3,5‐tri(*m*‐pyrid‐3‐yl‐phenyl)benzene (TmPyPB) (45 nm)/LiF (1 nm)/Al (100 nm) (Figure 6c). Device data are shown in Figure 6, and in Table 2. There is greenish‐blue EL with *λ*_max_ values, which are almost identical to their PL spectra in neat films (Table 1; Figure S28). The luminance of **DCz‐DPS‐TCz** (*L*_max_ 4080 cd m^−2^) is higher than **DCz‐DPS‐Cz** (*L*_max_ 2203 cd m^−2^), which is ascribed to less exciton quenching in the electric field (Figure 6a). For ten tests, **DCz‐DPS‐Cz** and **DCz‐DPS‐TCz** based devices achieved a maximum current efficiency (CE) of 62.0 and 65.9 cd A^−1^, and a maximum power efficiency (PE) of 51.6 and 59.2 lm W^−1^ (Figure 6b). Low turn‐on voltages of 3.1 and 3.0 V were obtained. **DCz‐DPS‐Cz**‐based devices exhibit an EQE_max_ of 23.3 %, and EQEs of 22.9 % and 18.7 % were obtained at 100 and 500 cd m^−2^. A high EQE_max_ of 24.0 % for **DCz‐DPS‐TCz** was obtained; the EQE was 23.6 % and 21.3 % at 100 and 500 cd m^−2^ (Figure 6c; Figure S29). The low efficiency roll‐off can originate from the short radiative lifetimes of the emitters. Moreover, although **DCz‐DPS‐Cz** devices exhibit more balanced hole‐ and electron‐transporting abilities at low current density, **DCz‐DPS‐TCz** devices show more stable hole‐ and electron‐transport over the operating voltages, which is also beneficial to reducing efficiency roll‐off. The *J*–*V* curves of the hole‐ and electron‐only devices are shown in Figure S30. These results exceed the current state‐of‐the‐art performance for any reported non‐doped macromolecular devices (Figure 6d).

**Table 2:** EL properties of solution‐processed non‐doped devices.

Device	*λ*_EL_ [nm]^[a]^	*V*_on_ [V]^[b]^	*CE*_max_ [cd A^−1^]^[c]^	*PE*_max_ [lm W^−1^]^[d]^	*EQE*_max/100/500_ [%]^[e]^	*CIE*_*(x,y)*_^[f]^
**A**	498	3.1	62.0	51.6	23.3/22.9/18.7	(0.23, 0.42)
**B**	500	3.0	65.9	59.2	24.0/23.6/21.3	(0.24, 0.45)

[a] The peak value of electroluminescence. [b] Turn‐on voltage at 1 cd m^−2^. [c] Maximum current efficiency. [d] Maximum power efficiency. [e] Maximum external quantum efficiency and external quantum efficiency at 100 cd m^−2^ and 500 cd m^−2^. [f] Coordinates of Commission Internationale de l’éclairage.

## Conclusion

The new TADF emitters **DCz‐DPS‐Cz** and **DCz‐DPS‐TCz** have a high *k*_RISC_≥2.6×10^6^ s^−1^ rate ascribed to the tiny Δ*E*_ST_≈30 meV without degenerate excited states. The ^3^LE state is ≥0.3 eV above the lowest ^3^CT state, which can also effectively mediate the spin flip from the triplet state to the singlet state, assuring a solely thermal equilibrium route for a fast RISC process, resulting in an efficient TADF performance. In addition, These dendronized emitters exhibit AEE properties, which could reduce the exciton quenching in aggregated states, and consequently reduce efficiency roll‐off in OLEDs. Non‐doped solution‐processed **DCz‐DPS‐Cz**‐based OLEDs have EQE_max_ values of 23.3 % and 22.9 % at 100 cd m^−2^. **DCz‐DPS‐TCz**‐based devices have EQE_max_ values of 24.0 % and 23.6 % at 100 cd m^−2^. The results provide a new perspective for the development of high‐efficiency TADF emitters and their non‐doped solution‐processable devices. Benefitting from the high fluorescence efficiency and excellent processability, these emitters can be envisaged in fluorescence imaging,[^90^, ^91^] optical temperature sensing,^[92]^ and ratiometric oxygen sensing.^[92]^

## Conflict of interest

The authors declare no conflict of interest.

1

## Supporting information

As a service to our authors and readers, this journal provides supporting information supplied by the authors. Such materials are peer reviewed and may be re‐organized for online delivery, but are not copy‐edited or typeset. Technical support issues arising from supporting information (other than missing files) should be addressed to the authors.

Supporting Information

## Data Availability

The data that support the findings of this study are available in the Supporting Information of this article.
