# Supplementary material for: Asymmetrical‐Dendronized TADF Emitters for Efficient Non‐doped Solution‐Processed OLEDs by Eliminating Degenerate Excited States and Creating Solely Thermal Equilibrium Routes
Source: Angew Chem Int Ed Engl. 2022 Jan 20;61(19):e202115140. doi: 10.1002/anie.202115140 (PMC9306820; doi:10.1002/anie.202115140)
Supplement: Supplementary file 1 — Supporting Information [file ANIE-61-0-s001.pdf]

Angewandte Chemie
Eine Zeitschrift der Gesellschaft Deutscher Chemiker

Supporting Information

Asymmetrical-Dendronized TADF Emitters for Efficient Non-doped Solution-Processed OLEDs by Eliminating Degenerate Excited States and Creating Solely Thermal Equilibrium Routes

C. Li, A. K. Harrison, Y. Liu, Z. Zhao, C. Zeng, F. B. Dias, Z. Ren*, S. Yan, M. R. Bryce**

Table of Contents

1. **Characterization**
2. **Device Fabrication and Characterization**
3. **Materials Synthesis**
4. **Supporting Figures**
5. **References**

1. Characterization. ^{1}H NMR and ^{13}C NMR spectra were recorded on a Bruker AV400 (400 MHz) spectrometer. Chemical shifts (δ) are given in parts per million (ppm) relative to tetramethylsilane (TMS; δ = 0) as the internal reference. ^{1}H NMR spectra data are reported as chemical shift, relative integral, multiplicity (s = singlet, d = doublet, m = multiplet), coupling constant (*J* in Hz), and assignment. UV−vis absorption spectra were recorded on a Hitachi U-2900 spectrophotometer. Thermogravimetric analysis (TGA) was performed with a METTLER TOLEDO TGA/DSC 1/1100SF instrument. The thermal stability of the samples under a nitrogen atmosphere was determined by measuring their weight loss while heating at a rate of 10 °C min^{-1} from 25 to 800 °C. Cyclic voltammetry (CV) was carried out in nitrogen-purged acetonitrile at room temperature with a CHI 660E voltammetric analyzer. Tetrabutylammonium hexafluorophosphate ($TBAPF_6$) (0.1 M) was used as the supporting electrolyte. The conventional three-electrode configuration consisted of a glassy carbon working electrode, a platinum wire auxiliary electrode, and an Ag/AgCl pseudo-reference electrode with ferrocenium−ferrocene (Fc^+/Fc) as the external standard. Cyclic voltammograms were obtained at scan rate of 50 mV s^{-1}. The onset potential was determined from the intersection of two tangents drawn at the rising and background currents of the cyclic voltammogram. Phosphorescence, prompt fluorescence (PF), and delayed fluorescence (DF) spectra and decays were recorded using nanosecond gated luminescence and lifetime measurements (from 400 ps to 1 s) with either a high-energy pulsed Nd:YAG laser emitting at 355 nm (EKSPLA) or a N_2 laser emitting at 337 nm. Emission was focused onto a spectrograph and detected on a sensitive gated iCCD camera (Stanford Computer Optics 4 Picos) having subnanosecond resolution. PF/DF time-resolved measurements were performed by exponentially increasing the gate and delay times as described previously.[1] DFT and TD-DFT calculations were performed on Gaussian 09[2] on the nodes of a supercomputer (High performance computing platform, Beijing University of Chemical Technology). CCDC 2093520 contains the supplementary crystallographic data for this paper. These data can be obtained free of

charge from The Cambridge Crystallographic Data Centre via www.ccdc.cam.ac.uk/data_request/cif.

Device Fabrication and Characterization. The hole-injection material PEDOT:PSS (8000) and electron-transporting TmPyPB and hole transporting material poly(*N*-vinylcarbazole) (PVK) (Mw 136 600, Mn 56 400) were obtained from commercial sources. ITO-coated glass with a sheet resistance of 10 Ω per square was used as the substrate. Before device fabrication, the ITO-coated glass substrate was precleaned and exposed to UV-ozone for 2 min. PEDOT:PSS was then spin-coated onto the clean ITO substrate as a hole-injection layer. Then, PVK was spin-coated at 2000 rpm for 30 s from 10 mg mL^{-1} chlorobenzene solution onto PEDOT:PSS and baked at 120 °C for 15 min. Next a solution of the emitter in toluene was spin-coated (1 mg/mL; 2000 rpm) to form a 40 nm thick emissive layer and annealed at 80 °C for 30 min to remove the residual solvent. Finally, a 45 nm thick electron-transporting layer of TmPyPB was vacuum deposited, and a cathode composed of a 1 nm thick layer of LiF and aluminum (100 nm) was sequentially deposited onto the substrate through shadow masking with a pressure of 10^{-6} Torr. Deposition rates are 1–2 Å. s^{-1} for organic materials, 0.1 Å. s^{-1} for LiF, and 6 Å. s^{-1} for Al, respectively. The current density-voltage-luminance (*J-V-L*) characteristics of the devices were measured using a Keithley 2400 Source meter and a Keithley 2000 Source multimeter. The EL spectra were recorded using a JYSPEX CCD3000 spectrometer. The EQE values were calculated from the luminance, current density, and electroluminescence spectrum according to previously reported methods. All measurements were performed at room temperature under ambient conditions.

Synthesis. The synthesis of **DCz-DPS-Cz 6** is shown in Scheme S1.

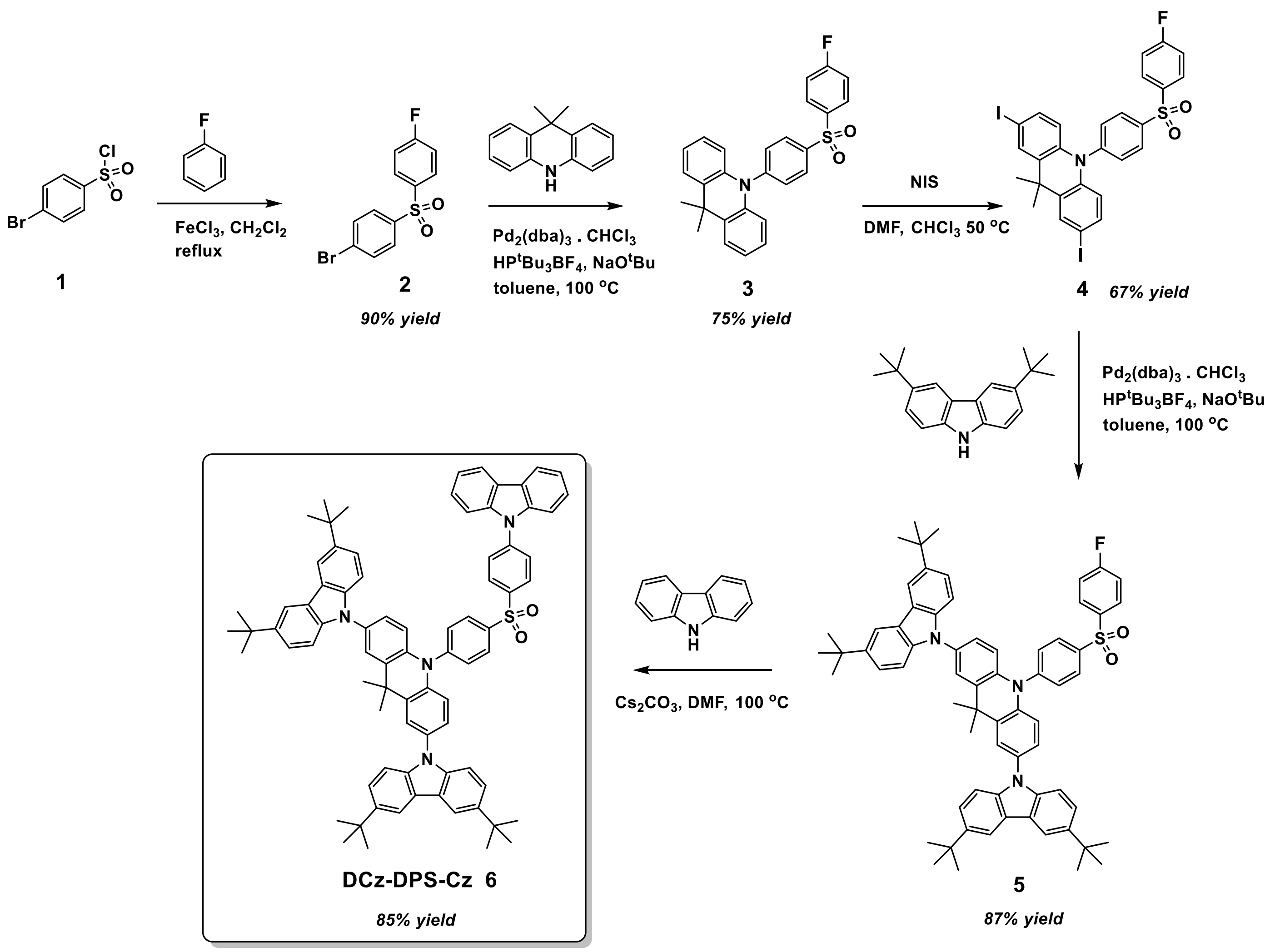

Scheme S1. Synthesis of **DCz-DPS-Cz 6**

1-bromo-4-((4-fluorophenyl)sulfonyl)benzene (compound **2**)[2] : 4-bromobenzenesulfonyl chloride **1** (5.00 g, 19.69 mmol) and fluorobenzene (2.84 g, 29.54 mmo1) were added into a 250 mL three-port flask and the mixture was dissolved in dichloromethane (20 mL). Ferric chloride (6.33 g, 39.38 mmol) was added and then the mixture was heated to 40 °C and stirred for 6 h. The mixture was cooled to room temperature, before adding dichloromethane (30 mL) followed by dilute hydrochloric acid (1 M, 50 mL) with stirring for 10 min. The organic layer was separated and the aqueous layer with was extracted three times with dichloromethane. The organic layers were combined, dried with anhydrous sodium sulfate, filtered and solvent removed to give **2** (5.54 g, 90% yield) as a white solid after vacuum drying. ^{1}H-NMR (400 MHz, $CDCl_3$) δ 8.00-7.90 (m, 1H), 7.84-7.74 (m, 1H), 7.72-7.61 (m, 1H), 7.23-7.15 (m, 1H).

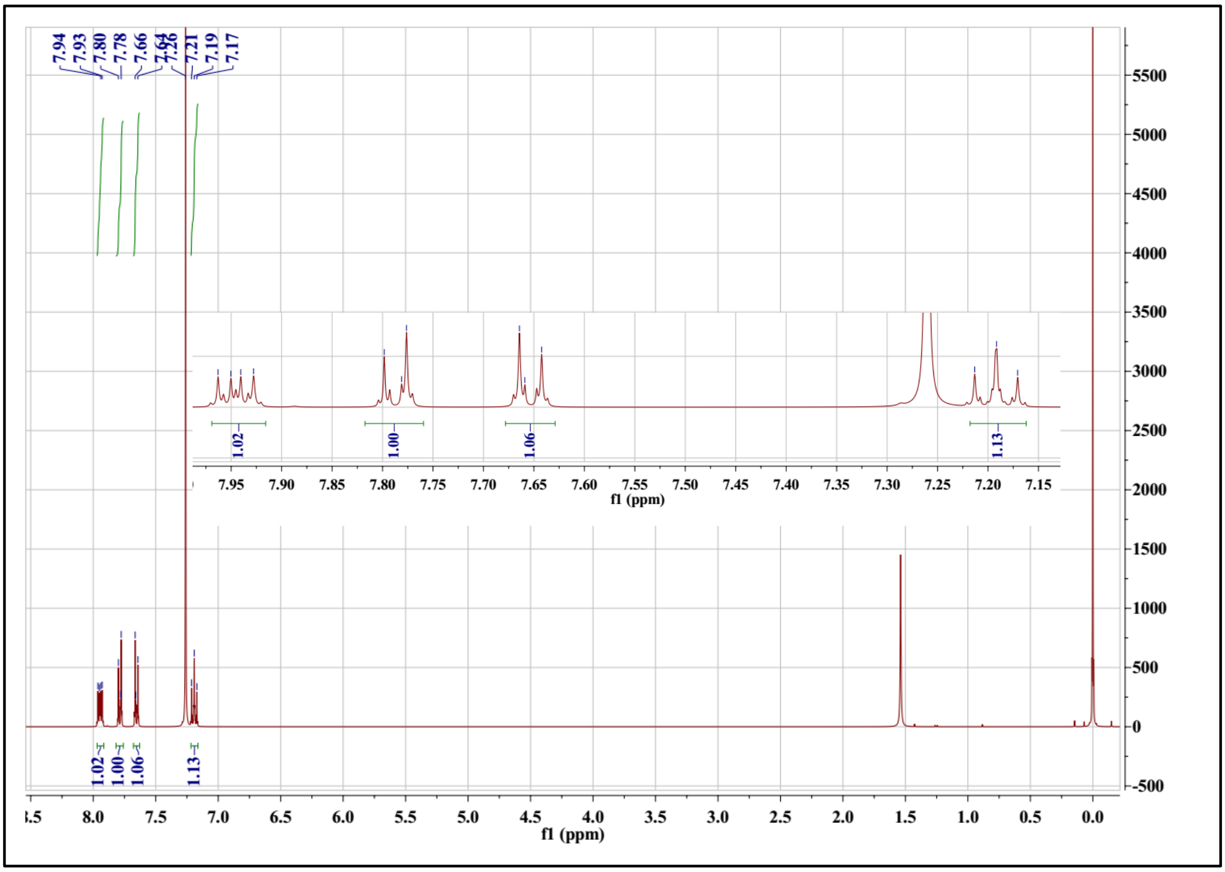

Fig. S1 ^{1}H-NMR spectrum of 1-bromo-4-((4-fluorophenyl)sulfonyl)benzene **2**

10-(4-((4-fluorophenyl)sulfonyl)phenyl)-9,9-dimethyl-9,10-dihydroacridine (compound **3**): A mixture of compound **2** (458 mg, 1.46 mmol), 9,9-dimethyl-10*H*-acridine (304 mg, 1.46 mmol), tris(dibenzylideneacetone)dipalladium ($Pd_2(dba)_3 \cdot CHCl_3$) (38 mg, 37 μmol), (t-Bu)$_3$PH-BF_4 (21 mg, 73 μmol) and sodium *tert*-butoxide (NaO^{t}Bu) (210 mg, 2.19 mmol) in toluene (20 mL) was stirred at 100 °C under argon for 24 h. After cooling to room temperature, the mixture was washed with brine and the organic phase was separated and dried with anhydrous sodium sulfate. After filtration and evaporation of the solvent, the product mixture was applied to a silica gel column using cyclohexane/dichloromethane (4/1 v/v) as eluent to give the crude product as green power. The powder was further crystallized from a mixture of hexane and CH_2Cl_2 to afford the pure product **3** (485 mg, 75% yield). ^{1}H-NMR (400 MHz, Acetone-D$_6$) δ 8.30 (d, J = 8.0 Hz, 2H), 8.21 (dd, J = 8.0, 5.2 Hz, 2H), 7.66 (d, J = 8.0 Hz, 2H), 7.54 (d, J = 7.2 Hz, 2H), 7.47 (t, J = 8.5 Hz, 2H), 6.99 (p, J = 7.2 Hz, 4H), 6.29 (d, J = 7.8 Hz, 2H), 1.66 (s, 6H). ^{13}C-NMR (101 MHz, $CDCl_3$) δ 166.98, 164.43, 146.66, 140.30, 137.43, 131.68, 131.12, 130.17, 126.49, 125.44, 121.76, 117.02, 116.80, 115.02, 53.53, 36.25,

30.84. HRMS-ASAP-TOF+ (m/z) calcd. for $C_{27}H_{22}FNO_2S$ $[M+H]^+$: 444.1434; found: 444.1431. Anal. calcd for $C_{27}H_{22}FNO_2S$ (%): C, 73.12; H, 5.00; F, 4.28; N, 3.16; S, 7.23. found: C, 73.14; H, 4.96; N, 3.17; S, 7.21.

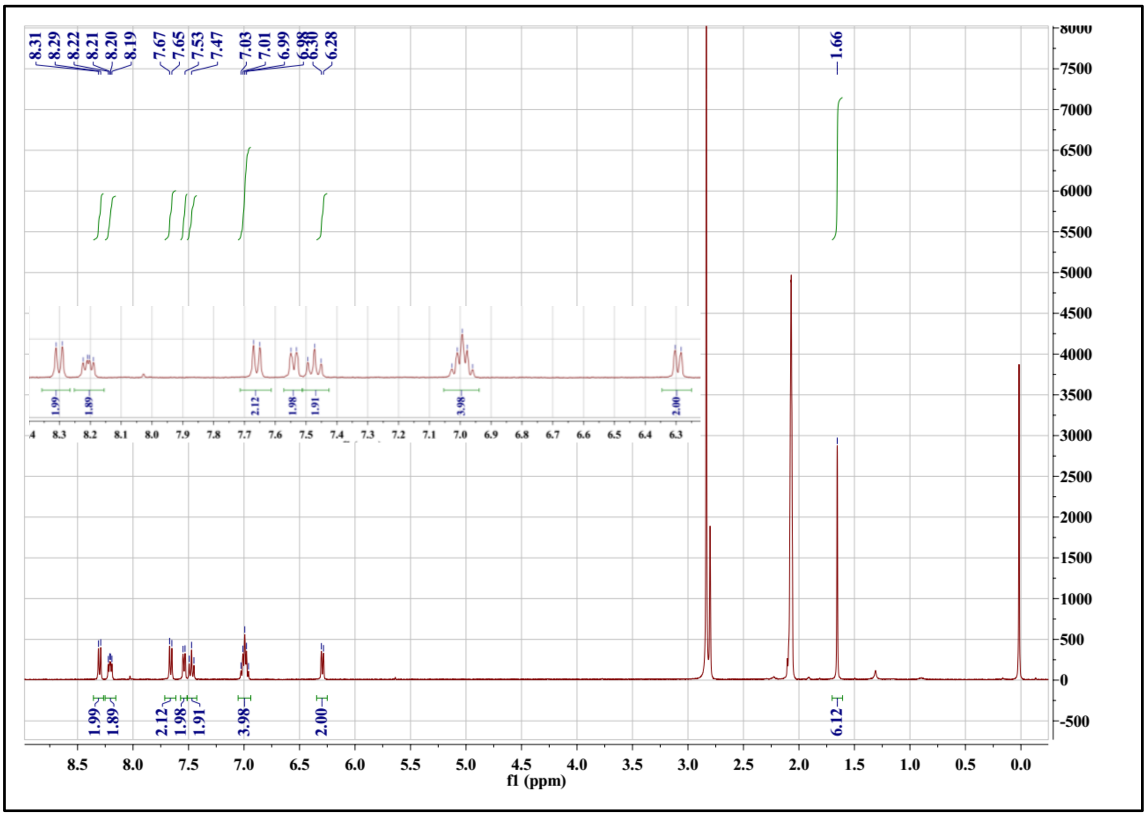

Fig. S2 ^{1}H-NMR spectrum of 10-(4-((4-fluorophenyl)sulfonyl)phenyl)-9,9-dimethyl-9,10-dihydroacridine **3**

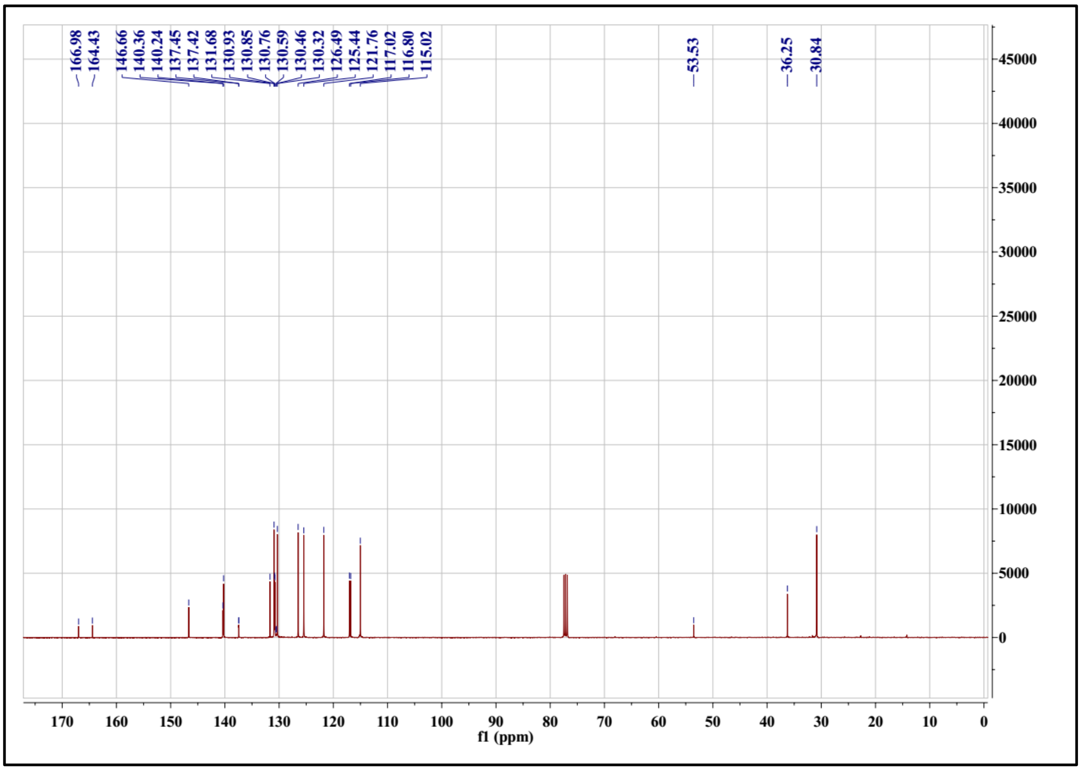

Fig. S3 ^{13}C-NMR spectrum of 10-(4-((4-fluorophenyl)sulfonyl)phenyl)-9,9-dimethyl-9,10-dihydroacridine **3**

Compound **4**: A solution of *N*-iodosuccinimide (472 mg, 2.1 mmol) in dry DMF (10 mL) was dropped into a solution of **3** (443 mg, 1.0 mmol) in dry chloroform (10 mL). The mixture was placed away from light and stirred at 50 °C for 48 hours. After that time, the reaction mixture was slowly cooled to room temperature, washed with aqueous sodium thiosulfate solution (3 x 50 mL). The mixture was extracted with CH_2Cl_2 and the organic phase was separated and dried over anhydrous Na_2SO_4. After filtration and removal of the solvent, the residue was purified by column chromatography (silica, hexane/CH_2Cl_2 v/v: 3:1) to give **4** as a green powder (466 mg, 67%). ^{1}H-NMR (400 MHz, $CDCl_3$) δ 8.27-8.17 (m, 1H), 8.15-8.05 (m, 1H), 7.74-7.65 (m, 1H), 7.51-7.44 (m, 1H), 7.35-7.21 (m, 2H), 5.97-5.87 (m, 1H). ^{13}C-NMR (101 MHz, $CDCl_3$) δ 167.09, 164.53, 145.24, 141.72, 139.61, 137.01, 135.41, 134.25, 132.88, 131.75, 131.52, 130.88, 130.49, 129.41, 127.60, 117.10, 116.87, 116.45, 84.31, 77.38, 77.07, 76.75, 53.48, 36.02, 31.62, 30.95, 29.73, 22.71, 14.17. HRMS-ASAP-TOF$^+$ (m/z) calcd. for $C_{27}H_{20}FI_2NO_2S$ $[M+H]^+$: 695.9366; found: 695.9409. Anal. calcd for $C_{27}H_{20}FI_2NO_2S$ (%): C, 46.64; H, 2.90; F, 2.73; N, 2.01; S, 4.61. found: C, 46.63; H, 2.93; N, 1.99; S, 4.62.

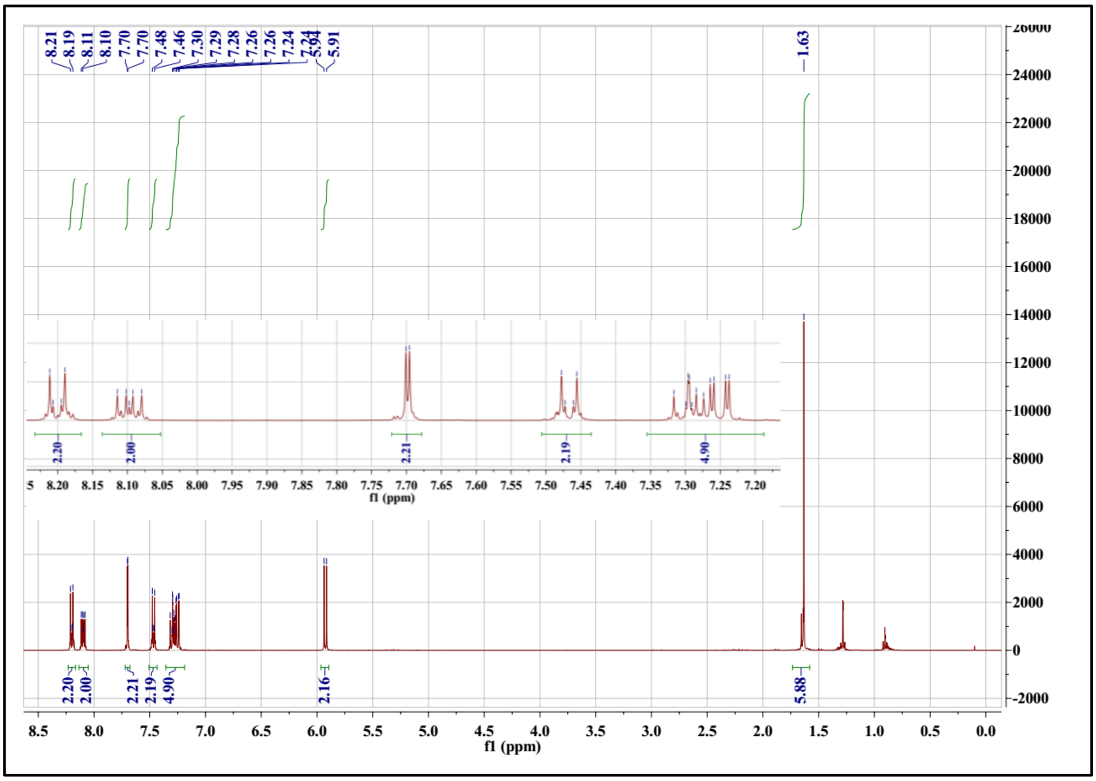

Fig. S4 ^{1}H-NMR spectrum of 2I-DMAC-DPS-F **4**

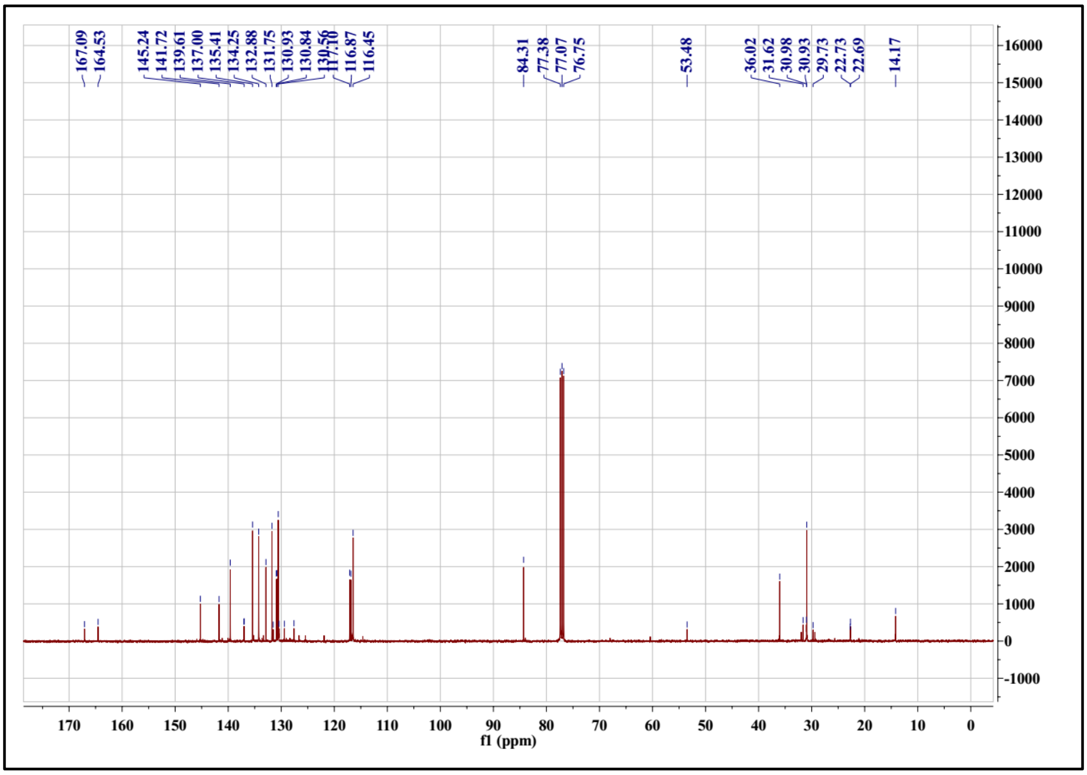

Fig. S5 ^{13}C-NMR spectrum of 2I-DMAC-DPS-F **4**

Compound **5**: A mixture of compound **4** (507.3 mg, 0.73 mmol), 3,6-di-*tert*-butyl-9*H*-carbazole (407.6 mg, 1.46 mmol), tris(dibenzylideneacetone)dipalladium ($Pd_2(dba)_3 \cdot CHCl_3$) (38 mg, 37 μmol), (*t*-Bu)$_3$PH-BF$_4$ (21 mg, 73 μmol) and sodium *tert*-butoxide (NaO^{t}Bu)(210 mg, 2.19 mmol) in toluene (20 mL) was stirred at 100 °C under argon for 24 h. After cooling to room temperature, the mixture was washed with brine and the organic phase was separated and dried with anhydrous sodium sulfate. After filtration and removing of the solvent, the product mixture was applied to a silica gel column using cyclohexane/dichloromethane 2/1 (v/v) as eluent to give the crude product as a green power. The powder was crystallized from a mixture of hexane and CH_2Cl_2 to afford the pure product **5** (634 mg, 87% yield). ^{1}H-NMR (400 MHz, Acetone-D$_6$) δ 8.45 (d, *J* = 7.8 Hz, 1H), 8.35-8.22 (m, 3H), 7.95 (d, *J* = 8.0 Hz, 1H), 7.82 (s, 1H), 7.51 (dd, *J* = 13.9, 8.6 Hz, 3H), 7.32 (t, *J* = 9.3 Hz, 3H), 6.59 (d, *J* = 8.8 Hz, 1H), 1.88 (s, 3H), 1.46 (s, 16H). ^{13}C-NMR (101 MHz, $CDCl_3$) δ 146.09, 143.00, 142.65, 141.46, 139.90, 139.51, 138.89, 132.18, 131.85, 130.91, 129.95, 127.89, 127.24, 126.48, 125.84, 125.55, 125.18, 124.47, 124.08, 123.58, 123.16, 121.10, 120.63, 116.31, 115.44, 109.59, 109.04, 36.69, 34.76, 34.25, 32.01, 31.47, 30.34, 29.73. HRMS-ASAP-TOF$^+$ (m/z) calcd. for $C_{67}H_{68}FN_3O_2S$ [M+H]$^+$: 998.5095;

found: 998.4938. Anal. calcd for $C_{67}H_{68}FN_3O_2S$ (%): C, 80.61; H, 6.87; N, 4.21; S, 3.21. found: C, 80.58; H, 6.89; N, 4.22; S, 3.22.

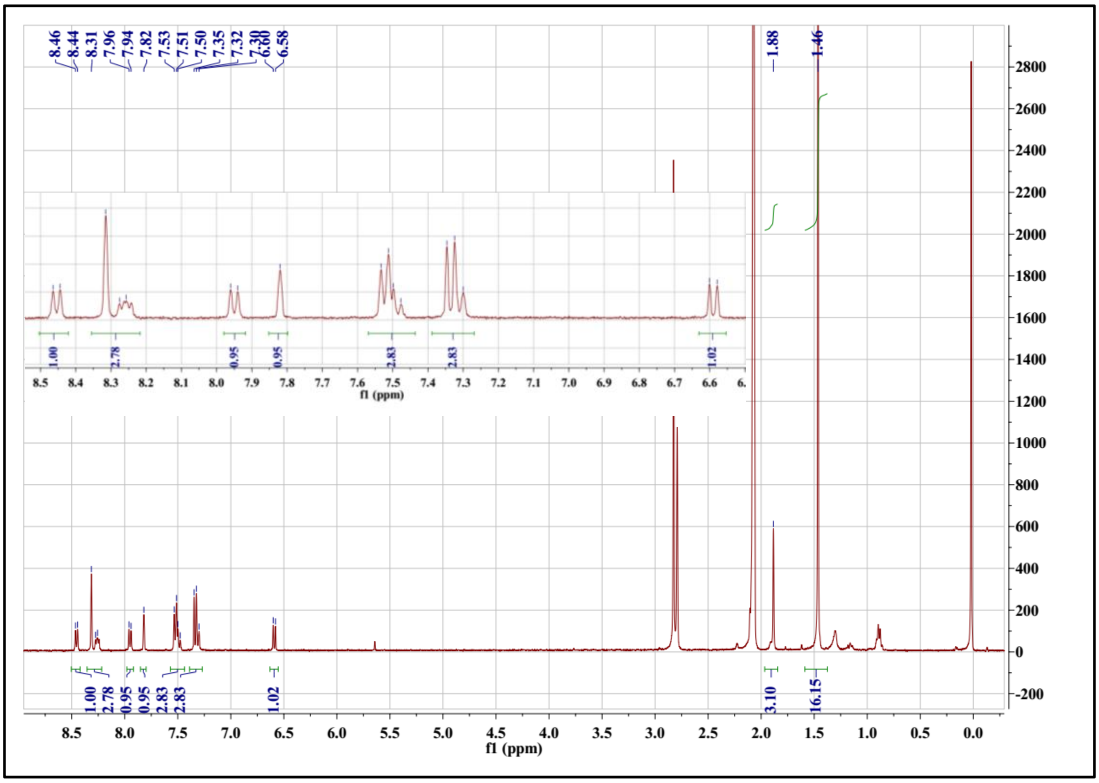

Fig. S6 ¹H-NMR spectrum of 2Cz-DMAC-DPS-F **5**

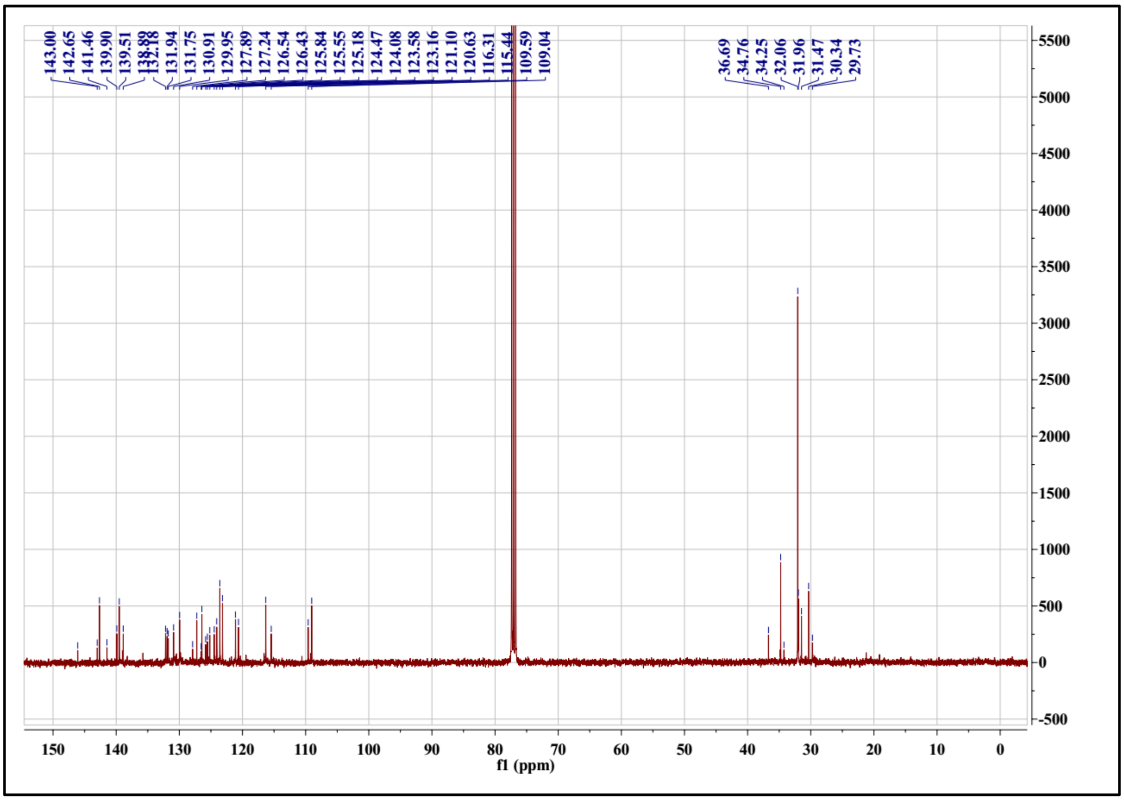

Fig. S7 ¹³C-NMR spectrum of 2Cz-DMAC-DPS-F **5**

DCz-DPS-Cz (**6**): A mixture of **5** (997.5 mg, 1.0 mmol), 9*H*-carbazole (167 mg, 1.0 mmol) and Cs_2CO_3 (391 mg, 1.2 mmol) was added to *N,N*-dimethylformamide (10 mL). The reaction was heated at 100 °C under nitrogen for 24 h. After cooling, the mixture was poured into water (200 mL). The crude product was filtered and purified by silica gel column chromatography, eluted with cyclohexane/dichloromethane 1/1 (v/v). The product was recrystallized from ethyl acetate to give **6** as a green product (973 mg, 85%). ^{1}H-NMR (400 MHz, $CDCl_3$) δ 8.49-8.41 (m, 1H), 8.39-8.32 (m, 2H), 8.30 (dd, J = 11.1, 6.4 Hz, 1H), 8.22-8.14 (m, 5H), 7.94-7.76 (m, 4H), 7.71 (dd, J = 13.0, 2.2 Hz, 2H), 7.60-7.41 (m, 9H), 7.41-7.20 (m, 8H), 7.09-6.96 (m, 2H), 6.50 (dd, J = 13.4, 5.7 Hz, 2H), 1.84-1.71 (m, 6H), 1.61-1.39 (m, 36H). ^{13}C-NMR (101 MHz, $CDCl_3$) δ 146.09, 143.00, 142.65, 141.46, 139.90, 139.51, 138.89, 132.18, 131.85, 130.91, 129.95, 127.89, 127.24, 126.48, 125.84, 125.55, 125.18, 124.47, 124.08, 123.58, 123.16, 121.10, 120.63, 116.31, 115.44, 109.59, 109.04, 36.69, 34.76, 32.01, 31.47, 30.34, 29.73. MALDI-MS$^+$ (m/z) calcd. for $C_{79}H_{76}N_4O_2S$ $[M]^+$: 1144.5; found: 1144.4. Anal. calcd for $C_{79}H_{76}N_4O_2S$ (%): C, 82.83; H, 6.69; N, 4.89; S, 2.80. found: C, 82.85; H, 6.71; N, 4.87; S, 2.78.

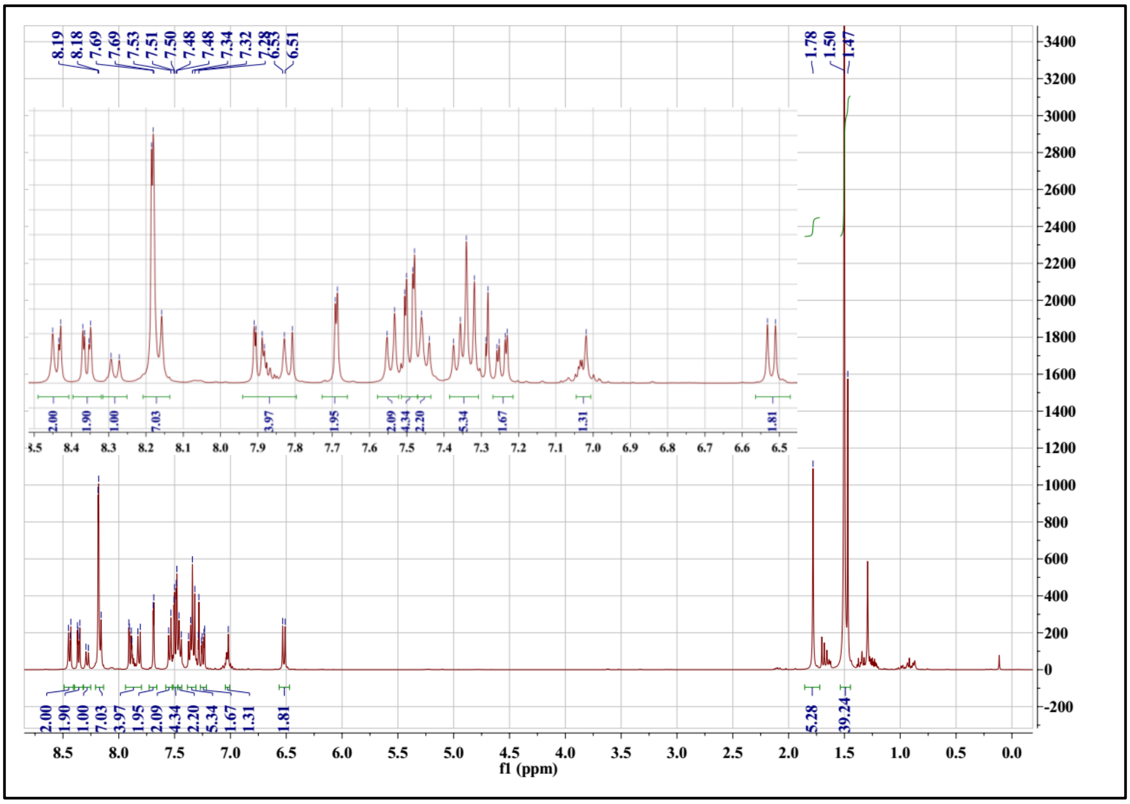

Fig. S8 ^{1}H NMR spectrum of DCz-DPS-Cz **6**

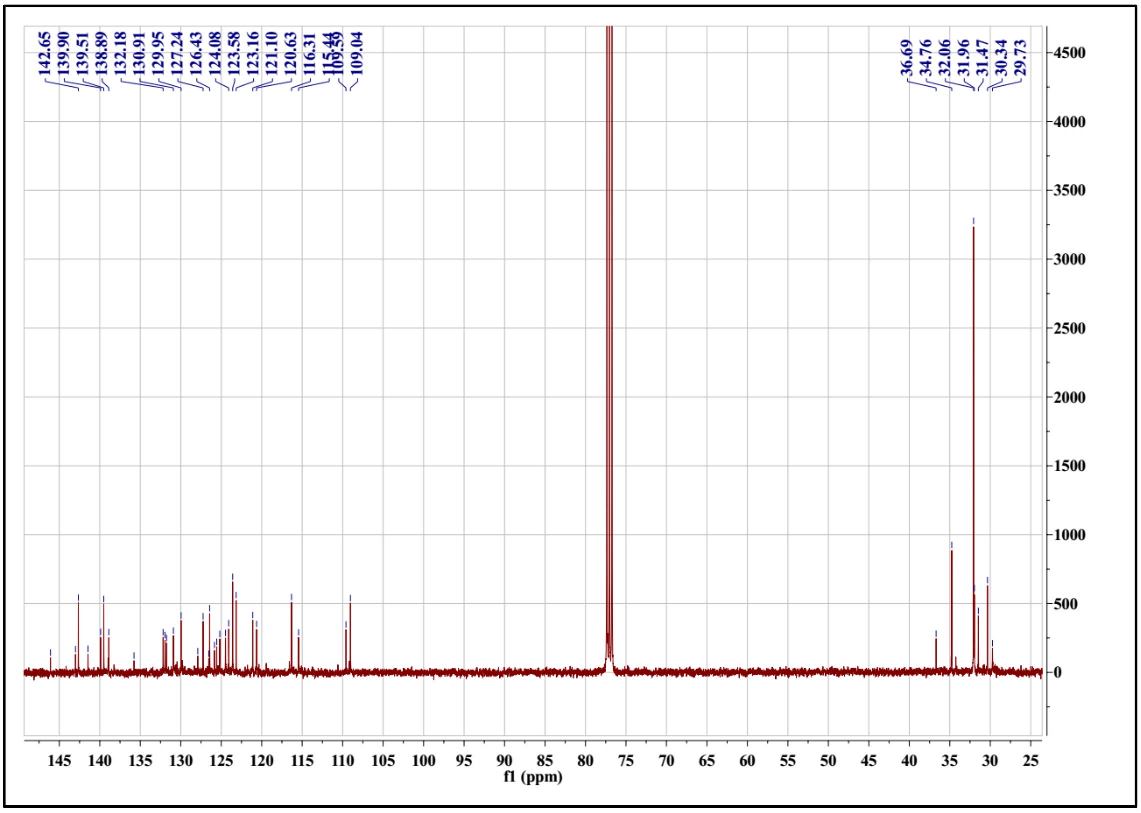

Fig. S9 ^{13}C NMR spectrum of DCz-DPS-Cz **6**

The synthesis of **DCz-DPS-TCz 13** is shown in Scheme S2.

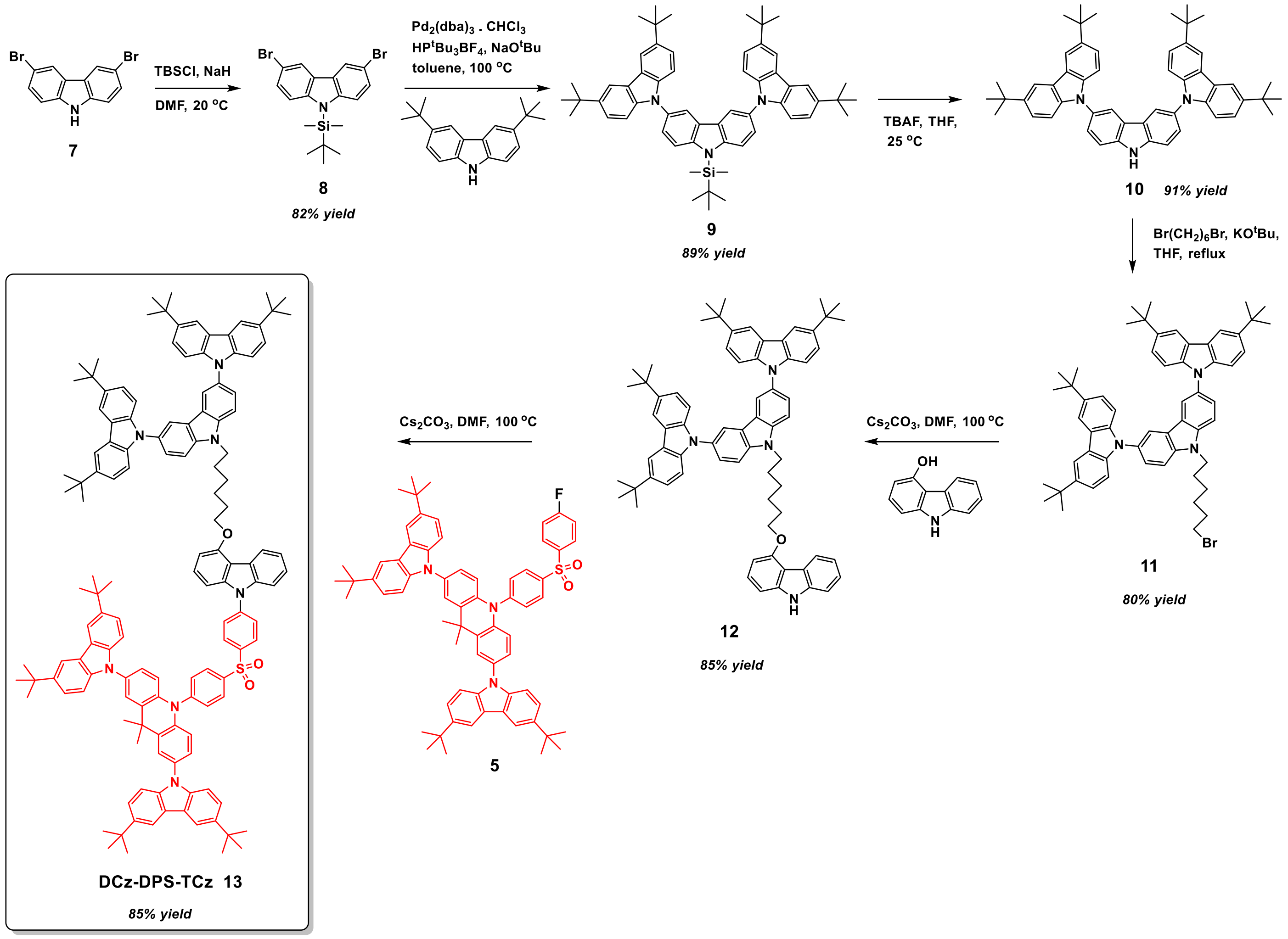

Scheme S2. The synthesis of **DCz-DPS-TCz 13.**

3,6-dibromo-9-(*tert*-butyldimethylsilyl)-9*H*-carbazole. Compound **8**: 3,6-dibromo-9*H*-carbazole **7** (9.7 g, 30.0 mmol) was dissolved in dry DMF and then NaH (1.08 g, 45.0 mmol) was added in batches at room temperature. Next, a solution of *tert*-butyldimethylchlorosilane (6.8 g, 45.0 mmol) in dry DMF (50 mL) was added dropwise. After stirring for 8 h, the mixture was poured into water and filtered to give the crude product, which was then purified by silica gel column chromatography (dichloromethane/hexane = 1:10, v/v) to give the pure product **8** as a white solid (10.8 g, 82% yield). ^{1}H-NMR (400 MHz, $CDCl_3$) δ 8.16-8.11 (m, 2H), 7.51-7.45 (m, 4H), 1.09-0.98 (m, 9H), 0.80-0.70 (m, 6H). ^{13}C-NMR (101 MHz, $CDCl_3$) δ 144.08, 128.69, 127.13, 122.76, 115.57, 112.89, 26.47, 25.69, 20.56, 1.28. HRMS-ASAP-TOF^{+} (m/z) calcd. for $C_{18}H_{21}Br_2NSi$ $[M+H]^{+}$: 437.9888; found: 437.9907. Anal. calcd for $C_{18}H_{21}Br_2NSi$ (%): C, 49.22; H, 4.82; N, 3.19. found: C, 49.24; H, 4.83; N, 3.20.

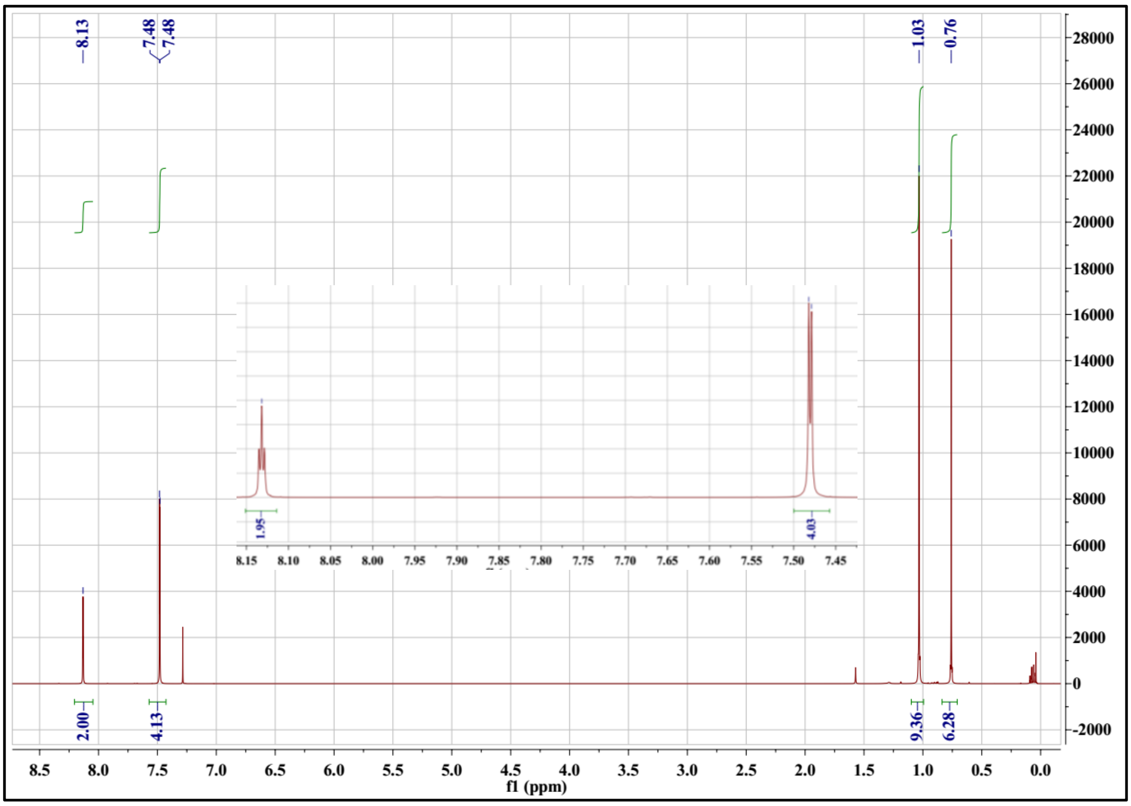

Fig. S10 ^{1}H NMR spectrum of 3,6-dibromo-9-(*tert*-butyldimethylsilyl)-9*H*-carbazole **8**

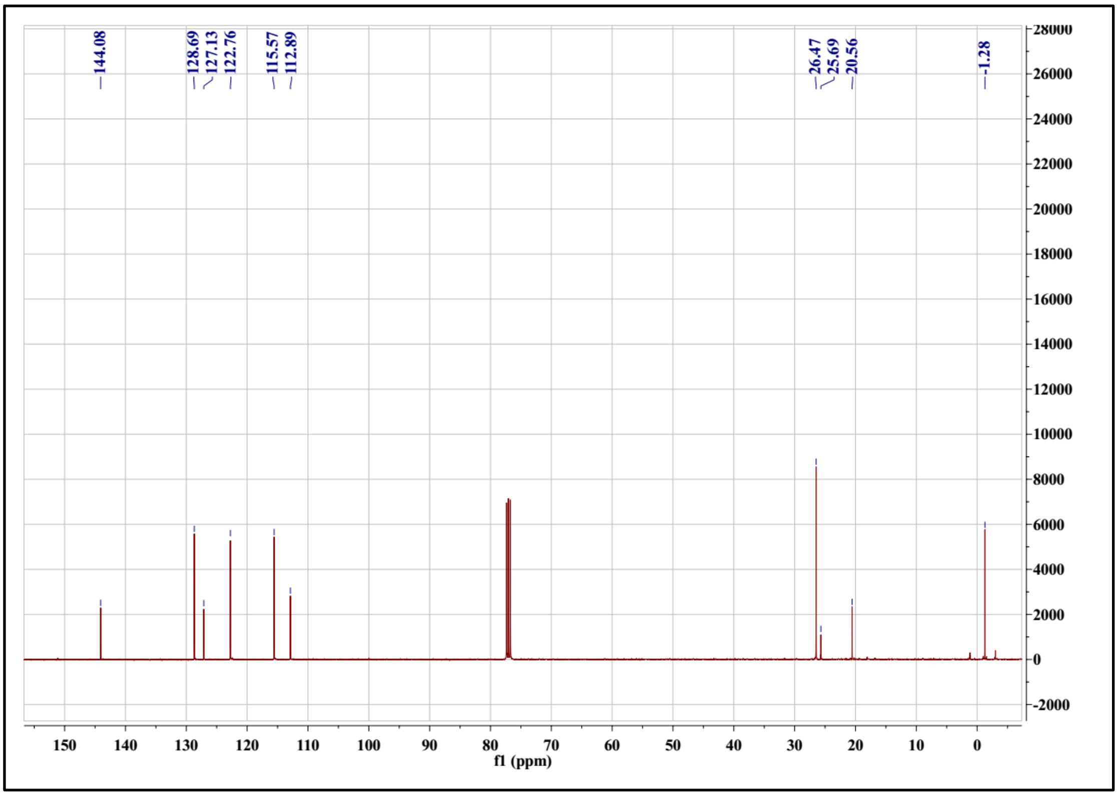

Fig. S11 ^{13}C NMR spectrum of 3,6-dibromo-9-(*tert*-butyldimethylsilyl)-9*H*-carbazole **8**

Compound 9: A mixture of compound **8** (319 mg, 0.73 mmol), 3,6-di-*tert*-butyl-9*H*-carbazole (408 mg, 1.46 mmol), tris(dibenzylideneacetone)dipalladium ($Pd_2(dba)_3 \cdot CHCl_3$) (38 mg, 37 µmol), (t-Bu)$_3$PH-BF_4 (21 mg,

73 μmol) and sodium *tert*-butoxide (NaO^tBu) (210 mg, 2.19 mmol) in toluene (20 mL) was stirred at 100 °C under argon for 24 h. After cooling to room temperature, the mixture was washed with brine and the organic phase was separated and dried with anhydrous sodium sulfate. After filtration and removal of the solvent, the product mixture was applied to a silica gel column using cyclohexane/dichloromethane (8/1 v/v) as eluent to give the crude product as white power. The powder was further crystallized from a mixture of hexane and CH_2Cl_2 to afford the pure product **9** (284 mg, 89% yield) as a white powder. ^{1}H-NMR (400 MHz, $CDCl_3$) δ 8.17 (d, J = 1.5 Hz, 6H), 7.84 (d, J = 8.8 Hz, 2H), 7.57 (dd, J = 8.8, 2.2 Hz, 2H), 7.47 (dd, J = 8.7, 1.9 Hz, 4H), 7.36 (d, J = 8.6 Hz, 4H), 1.48 (s, 36H), 1.21 (s, 9H), 0.91 (s, 6H). ^{13}C-NMR (101 MHz, $CDCl_3$) δ 144.72, 142.58, 140.22, 130.66, 127.21, 125.17, 123.64, 123.23, 118.72, 116.27, 115.29, 109.31, 34.84, 32.18, 31.72, 26.74, 22.79, 20.79, 14.27, 1.05. HRMS-ASAP-TOF$^+$ (m/z) calcd. for $C_{58}H_{69}N_3Si$ $[M+H]^+$: 836.5339; found: 836.5356. Anal. calcd for $C_{58}H_{69}N_3Si$ (%): C, 83.30; H, 8.32; N, 5.02. found: C, 83.28; H, 8.34; N, 5.01.

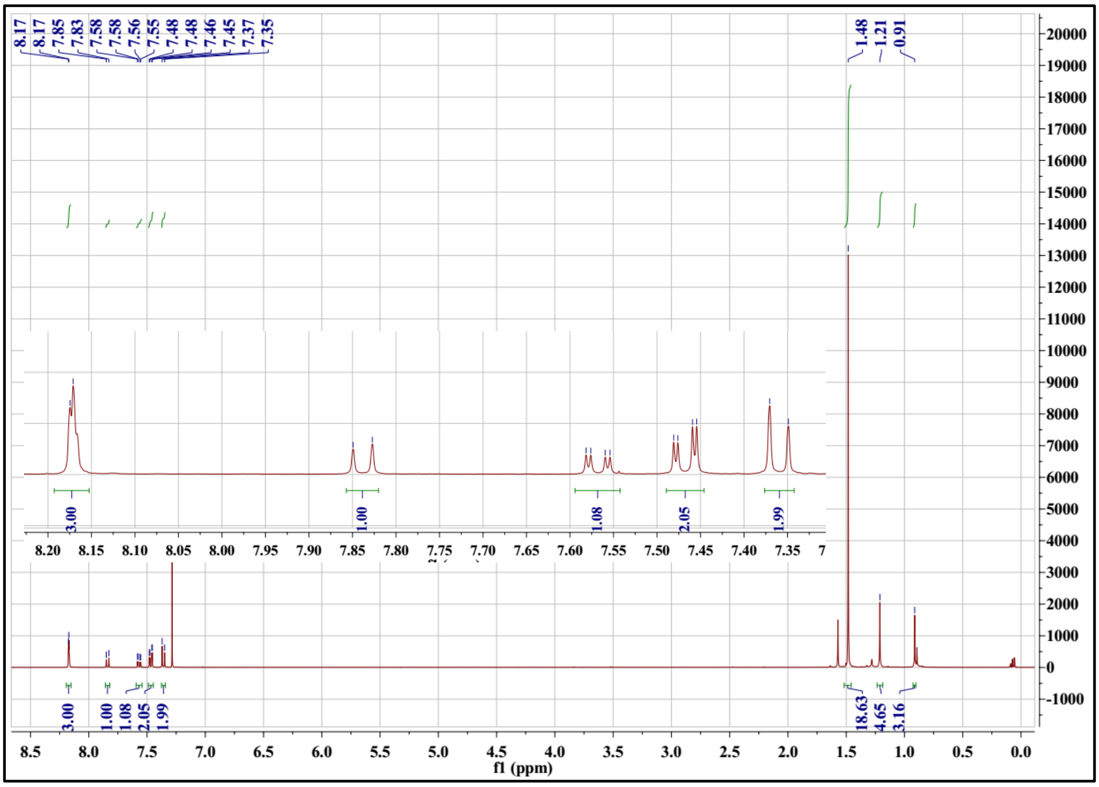

Fig. S12 ^{1}H-NMR spectrum of 3,3'',6,6''-tetra-*tert*-butyl-9'-(*tert*-butyldimethylsilyl)-9'*H*-9,3':6',9''-tercarbazole **9**

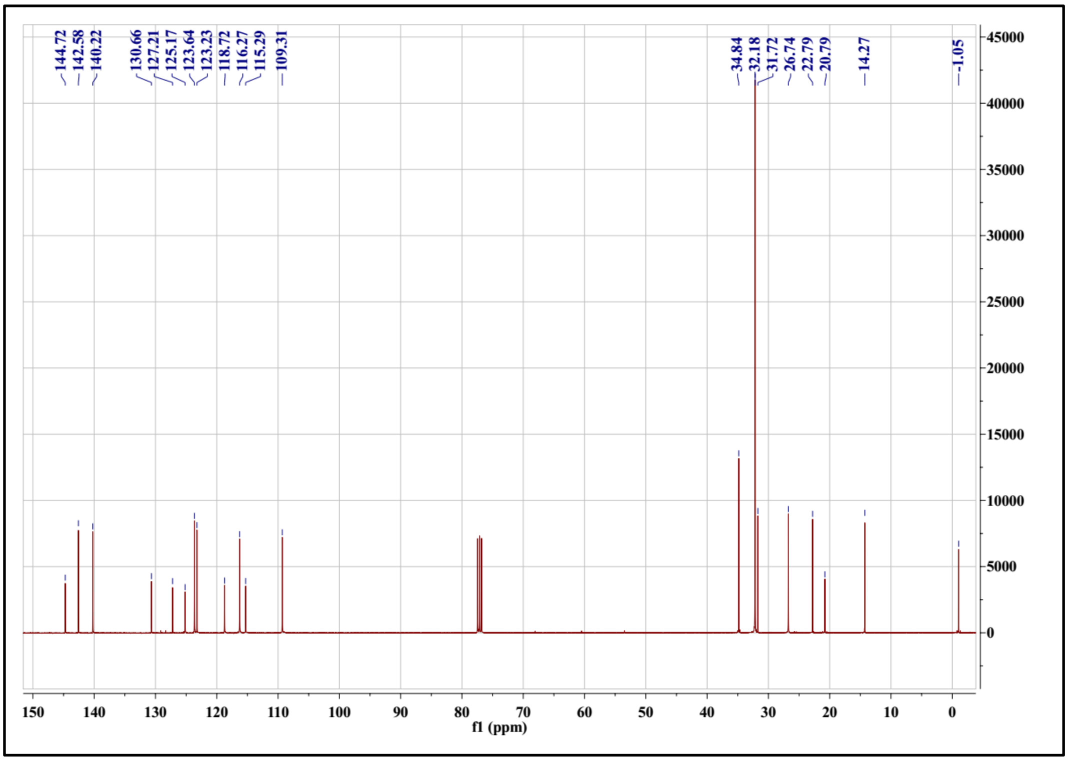

Fig. S13 ^{13}C-NMR spectrum of 3,3'',6,6''-tetra-*tert*-butyl-9'-(*tert*-butyldimethylsilyl)-9'*H*-9,3':6',9''-tercarbazole **9**

Compound **10**[3]: Compound **9** (4.37 g, 10 mmol) was dissolved in dry THF (50 mL), and then tetrabutylammonium fluoride (2.62 g, 10 mmol) was added. The mixture was stirred at 25 °C for 4 h, followed by pouring into water and filtration. The solid residue was collected and applied onto a silica gel column eluted with dichloromethane/hexane 1/3, v/v to give the product **10** as a white solid (6.57 g, 91% yield). ^{1}H-NMR (400 MHz, $CDCl_3$) δ 8.45 (s, 1H), 8.22-8.14 (m, 6H), 7.71 (d, *J* = 8.5 Hz, 2H), 7.63 (dd, *J* = 8.5, 2.0 Hz, 2H), 7.47 (dd, *J* = 8.6, 1.9 Hz, 4H), 7.34 (d, *J* = 8.6 Hz, 4H), 1.51-1.47 (m, 36H).

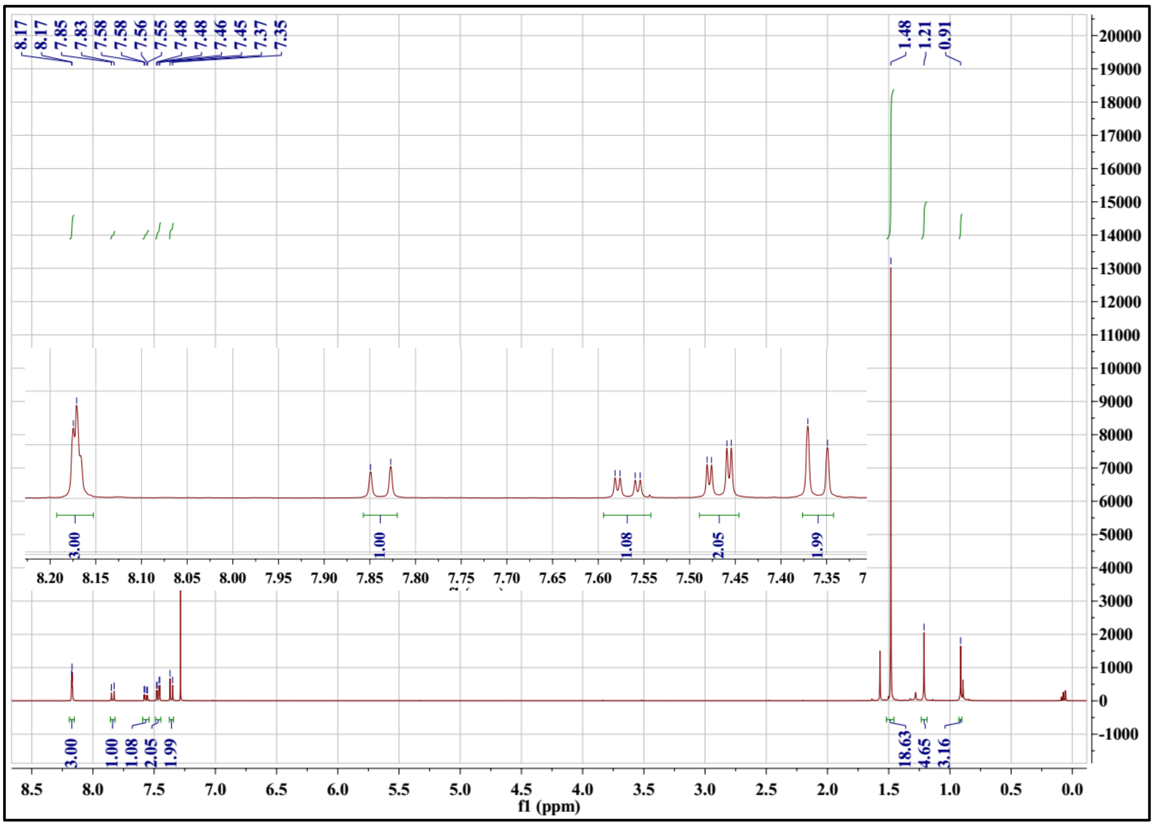

Fig. S14 ^{1}H-NMR spectrum of 3,3'',6,6''-tetra-*tert*-butyl-9'*H*-9,3':6',9''-tercarbazole **10**

Compound **11**: To a solution of compound **10** (3.6 g, 5.0 mmol) in dry THF (10 mL) was added KO^tBu (0.706 g, 6.3 mmol) in several portions. The resulting solution was stirred for 10 min at room temperature, and then added dropwise to a refluxing solution of 1,6-dibromohexane (4.5 mL, 29.2 mmol) in THF (10 mL). After refluxing for 24 h, the reaction mixture was cooled to room temperature and quenched by adding several drops of water to destroy the excessive KO^tBu. The THF solvent and excess 1,6-dibromohexane were removed by rotatory evaporation and vacuum distillation, respectively. The residual product was dissolved in CH_2Cl_2, washed with water for three times, the organic layer was dried over anhydrous Na_2SO_4, filtered and concentrated for flash column chromatography on silica (eluent CH_2Cl_2/petroleum ether 1:10 v/v), affording compound **11** as a white solid (3.5 g 80% yield). ^{1}H-NMR (400 MHz, $CDCl_3$) δ 8.19 (ddd, J = 2.7, 2.3, 1.2 Hz, 6H), 7.69-7.63 (m, 4H), 7.47 (dd, J = 8.7, 2.0 Hz, 4H), 7.37-7.30 (m, 4H), 4.55-4.49 (m, 2H), 3.47 (dd, J = 6.8, 2.5 Hz, 2H), 3.49-3.45 (m, 2H), 3.52-3.42 (m, 4H), 2.11 (dd, J = 12.8, 5.6 Hz, 2H), 1.93 (ddd, J = 13.9, 12.3, 6.5 Hz, 2H), 1.63 (dt, J = 12.5, 3.4 Hz, 4H), 1.52-1.46 (m, 36H). ^{13}C-NMR (101 MHz, $CDCl_3$) δ 142.48,

140.24, 139.92, 129.90, 125.75, 123.48, 123.06, 119.49, 116.20, 109.89, 109.12, 34.75, 33.77, 32.62, 32.08, 28.01, 26.64. HRMS-ASAP-TOF$^+$ (m/z) calcd. for $C_{58}H_{66}BrN_3$ $[M+H]^+$: 884.4518; found: 884.4540. Anal. calcd for $C_{58}H_{66}BrN_3$ (%): C, 78.71; H, 7.52; N, 4.75. found: C, 78.69; H, 7.51; N, 4.76.

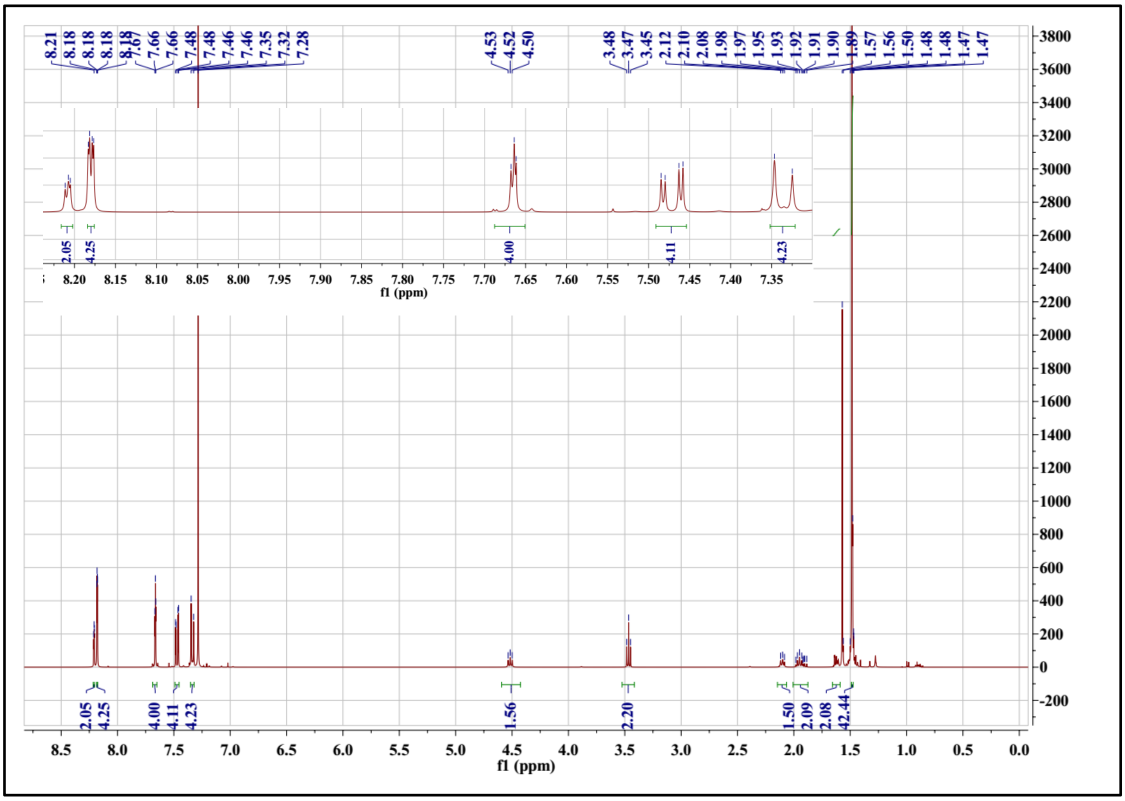

Fig. S15 ^{1}H-NMR spectrum of 9'-(6-bromohexyl)-3,3'',6,6''-tetra-*tert*-butyl-9'*H*-9,3':6',9''-tercarbazole **11**

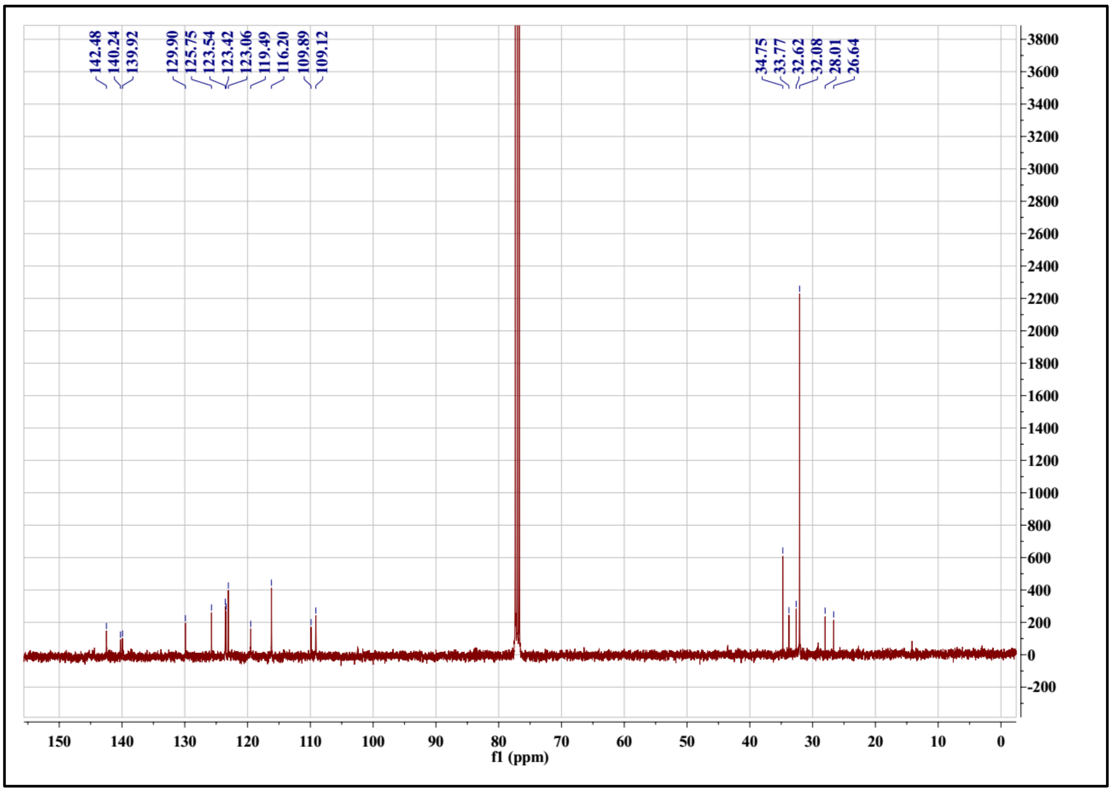

Fig. S16 ^{13}C-NMR spectrum of 9'-(6-bromohexyl)-3,3'',6,6''-tetra-*tert*-butyl-9'*H*-9,3':6',9''-tercarbazole **11**

Compound **12**: A mixture of **11** (883 mg, 1.0 mmol), 4-hydroxycarbazole (183 mg, 1.0 mmol) and Cs_2CO_3 (391 mg, 1.2 mmol) in *N,N*-dimethylformamide (10 mL)solution was heated at 100 ºC under nitrogen for 24 h. After cooling, the mixture was poured into water (200 mL). The crude product was filtered and purified by silica gel column chromatography, eluent CH_2Cl_2/petroleum ether (1:2 v/v). The product was recrystallized from ethyl acetate to give **12** as a green powder (839 mg, 85%). ^{1}H-NMR (400 MHz, $CDCl_3$) δ 8.37 (d, *J* = 7.8 Hz, 1H), 8.23-8.15 (m, 6H), 8.03 (s, 1H), 7.66-7.58 (m, 4H), 7.47 (dd, *J* = 8.7, 1.4 Hz, 4H), 7.40 (dd, *J* = 6.1, 1.1 Hz, 2H), 7.36-7.29 (m, 5H), 7.26 (d, *J* = 2.0 Hz, 1H), 7.03 (d, *J* = 7.8 Hz, 1H), 6.69 (d, *J* = 7.9 Hz, 1H), 4.52 (t, *J* = 7.2 Hz, 2H), 4.30 (t, *J* = 6.2 Hz, 2H), 2.21-2.05 (m, 4H), 1.88-1.79 (m, 2H), 1.77-1.70 (m, 2H), 1.49 (d, *J* = 0.4 Hz, 36H). ^{13}C-NMR (101 MHz, $CDCl_3$) δ 155.59, 142.45, 140.95, 140.26, 139.94, 138.71, 129.83, 126.72, 125.72, 124.96, 123.46, 123.19, 122.68, 119.53, 116.17, 112.72, 109.97, 109.18, 103.41, 101.09, 67.63, 43.57, 34.75, 32.08, 29.73, 29.33, 27.20, 26.30, 22.73. HRMS-ASAP-TOF$^+$ (m/z) calcd. for $C_{70}H_{74}N_4O$ $[M+H]^+$: 987.5941; found: 987.5934. Anal. calcd for $C_{70}H_{74}N_4O$ (%): C, 85.15; H, 7.55; N, 5.67. found: C, 85.17; H, 7.54; N, 5.66.

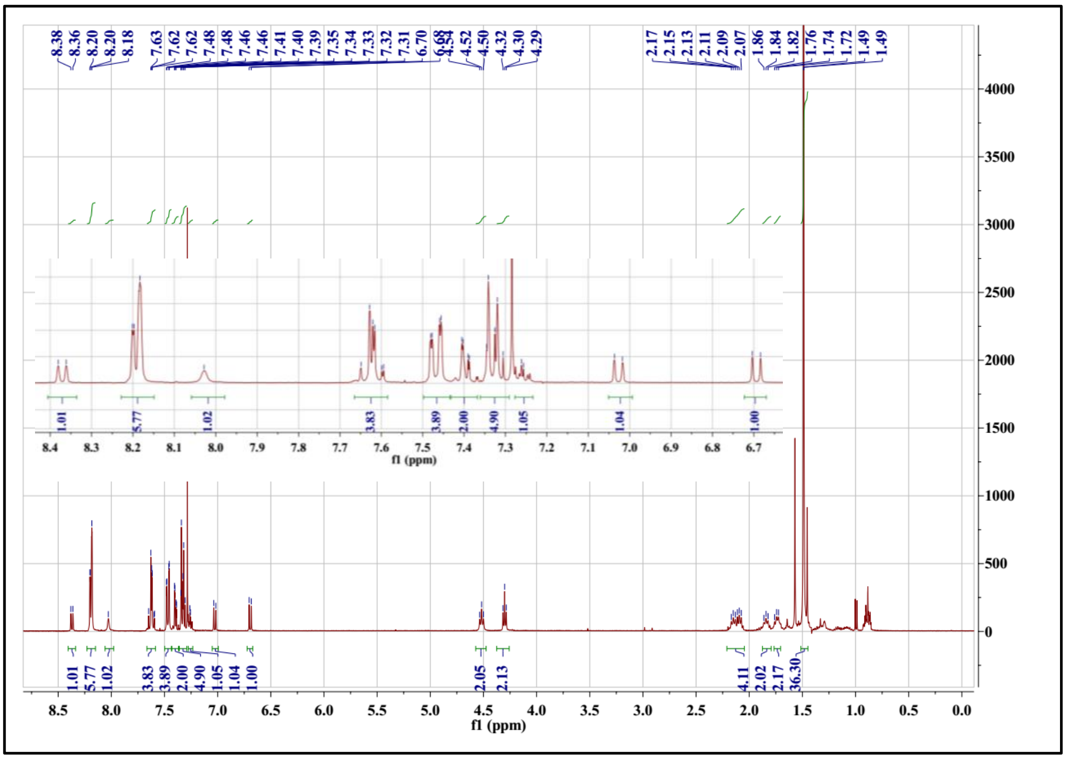

Fig. S17 ^{1}H-NMR spectrum of 9'-(6-((9*H*-carbazol-4-yl)oxy)hexyl)-3,3'',6,6''-tetra-*tert*-butyl-9'*H*-9,3':6',9''-tercarbazole **12**

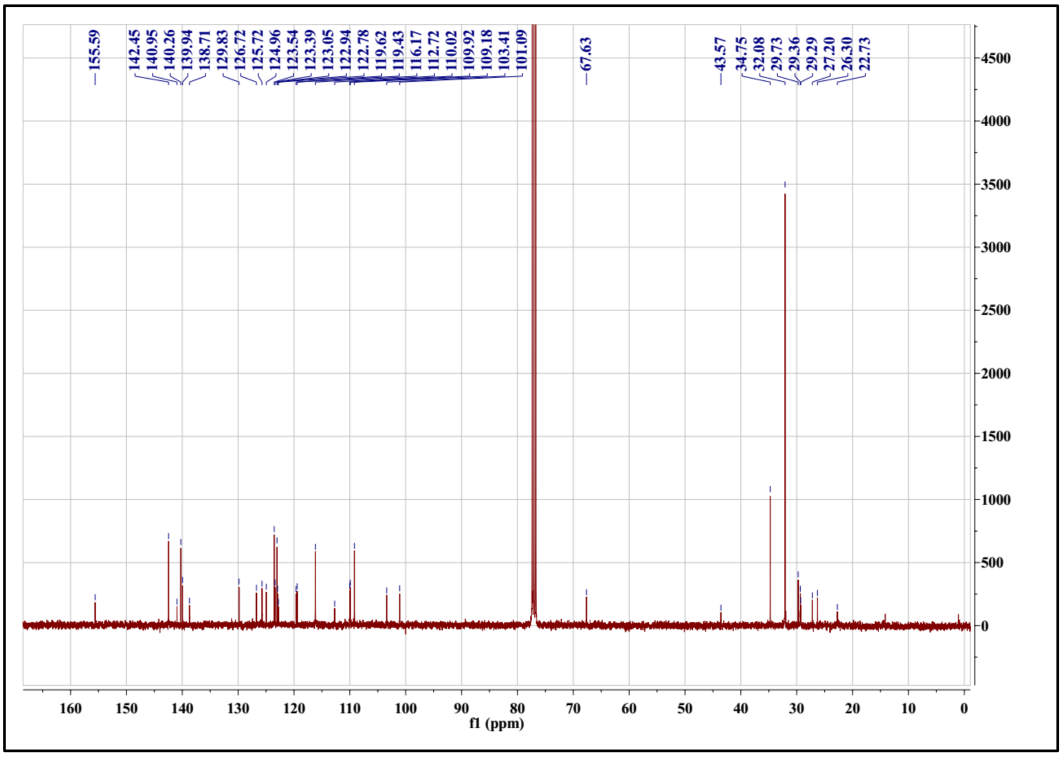

Fig. S18 ^{13}C-NMR spectrum of 9'-(6-((9*H*-carbazol-4-yl)oxy)hexyl)-3,3'',6,6''-tetra-*tert*-butyl-9'*H*-9,3':6',9''-tercarbazole **12**

DCz-DPS-TCz (**13**): A mixture of **5** (997.5 mg, 1.0 mmol), **12** (986.6 mg, 1.0 mmol) and Cs_2CO_3 (391 mg, 1.2 mmol) was added to *N,N*-dimethylformamide (10 mL). The solution was heated at 100 °C under argon for 24 h. After cooling, the mixture was poured into water (200 mL). The crude product was filtered and purified by silica gel column chromatography, eluent CH_2Cl_2/petroleum ether (1:1 v/v). The product was recrystallized with ethyl acetate to give **13** as a green powder (973 mg, 85%). ^{1}H-NMR (400 MHz, $CDCl_3$) δ 8.48-8.38 (m, 2H), 8.35-8.28 (m, 2H), 8.23-8.14 (m, 7H), 7.89-7.76 (m, 3H), 7.70-7.58 (m, 5H), 7.51-7.35 (m, 8H), 7.35-7.26 (m, 13H), 7.23 (dd, *J* = 8.7, 2.3 Hz, 2H), 7.09 (d, *J* = 8.4 Hz, 1H), 6.79 (d, *J* = 8.0 Hz, 1H), 6.50 (d, *J* = 8.7 Hz, 2H), 4.54 (t, *J* = 7.1 Hz, 2H), 4.32 (t, *J* = 6.2 Hz, 2H), 2.95 (dd, *J* = 29.0, 0.6 Hz, 2H), 2.25-2.07 (m, 4H), 1.94-1.82 (m, 2H), 1.81-1.70 (m, 7H), 1.56-1.37 (m, 56H), 1.37-1.25 (m, 5H), 1.03-0.73 (m, 6H). ^{13}C-NMR (101 MHz, $CDCl_3$) δ 155.77, 146.07, 143.06, 142.56, 141.42, 140.24, 139.95, 139.52, 139.21, 138.89,

132.05, 131.74, 130.90, 129.86, 127.37, 125.74, 125.49, 125.19, 124.47, 123.51, 123.11, 121.24, 119.47, 116.26, 115.46, 113.26, 109.91, 109.09, 102.75, 67.91, 43.57, 36.70, 34.76, 32.08, 31.48, 29.36, 27.24, 26.95, 26.31. MALDI-MS$^+$ (m/z) calcd. for $C_{137}H_{141}N_7O_3S$ [M]+: 1964.1; found: 1964.8. Anal. calcd for $C_{137}H_{141}N_7O_3S$ (%): C, 83.71; H, 7.23; N, 4.99. found: C, 83.73; H, 7.22; N, 5.02.

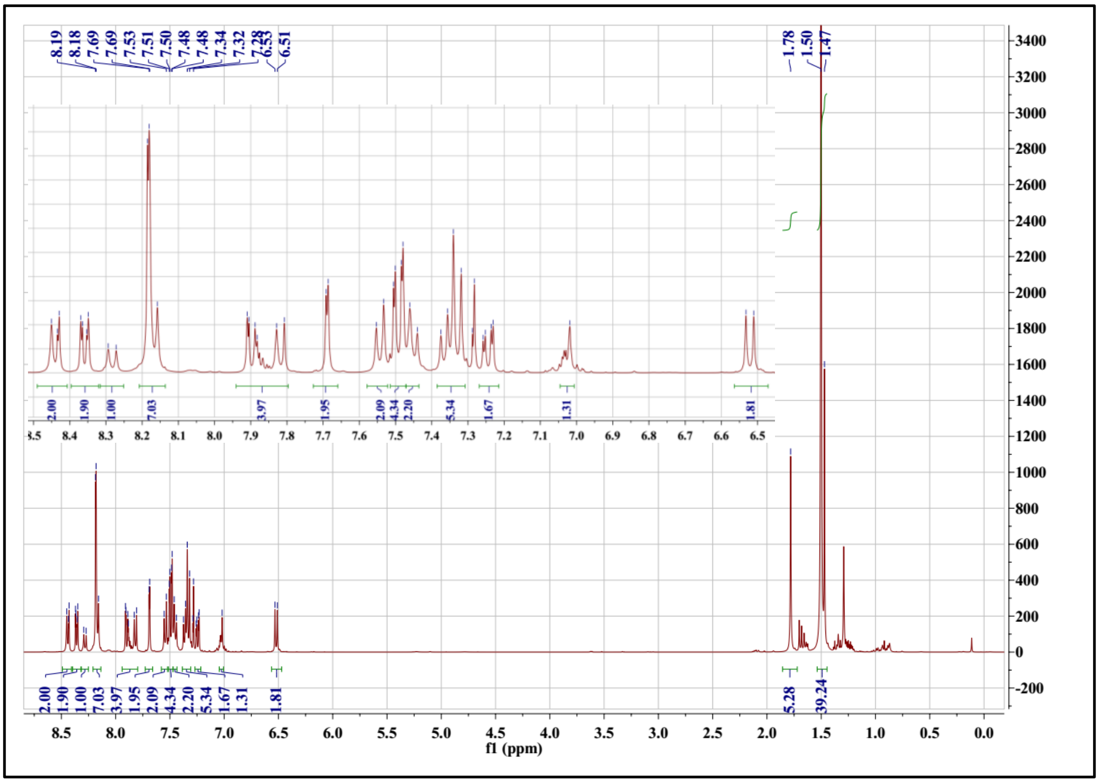

Fig. S19 ¹H-NMR spectrum of **DCz-DPS-TCz 13**

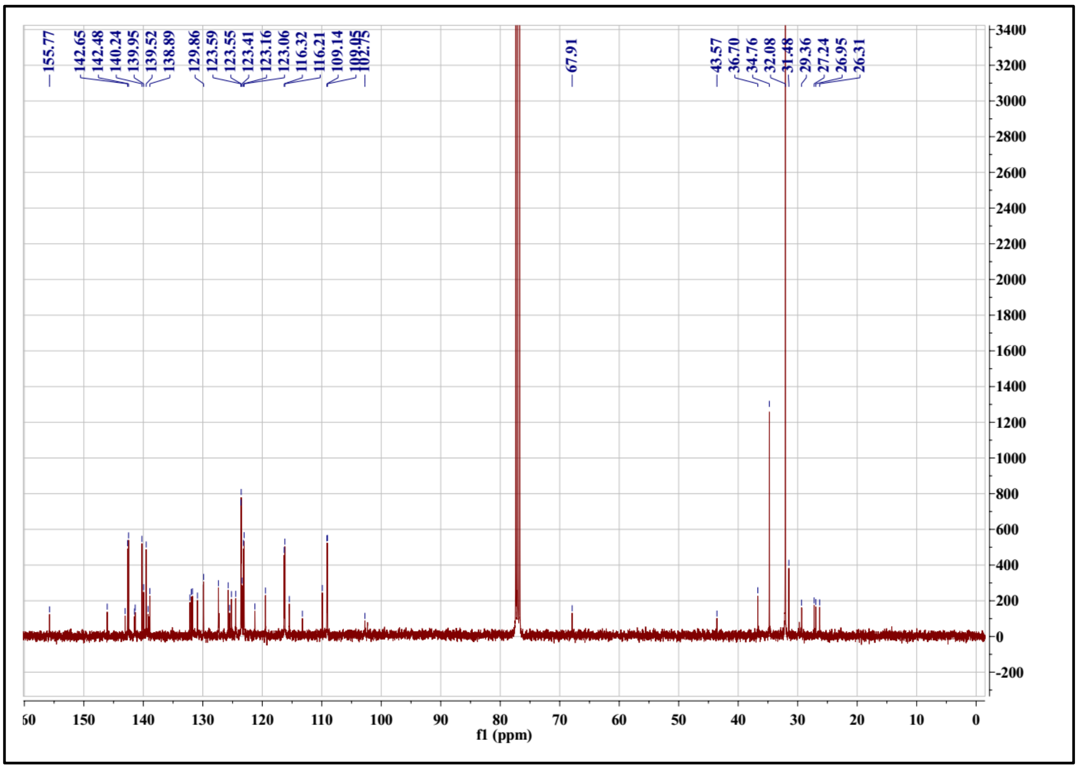

Fig. S20 ^{13}C-NMR spectrum of **DCz-DPS-TCz 13**

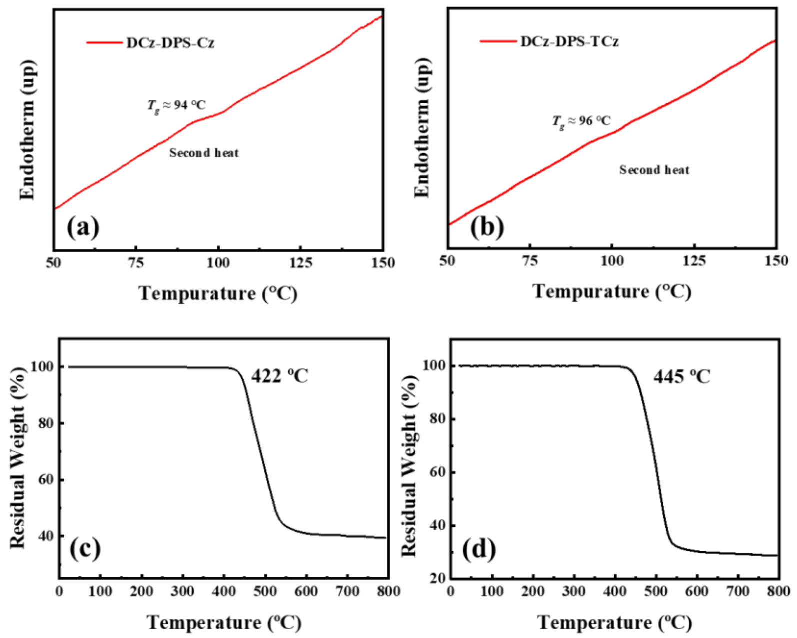

Fig. S21 DSC and TGA curves of (a, c) **DCz-DPS-Cz** and (b, d) **DCz-DPS-TCz** recorded at a heating rate of 10 °C min^{-1} from 20 °C.

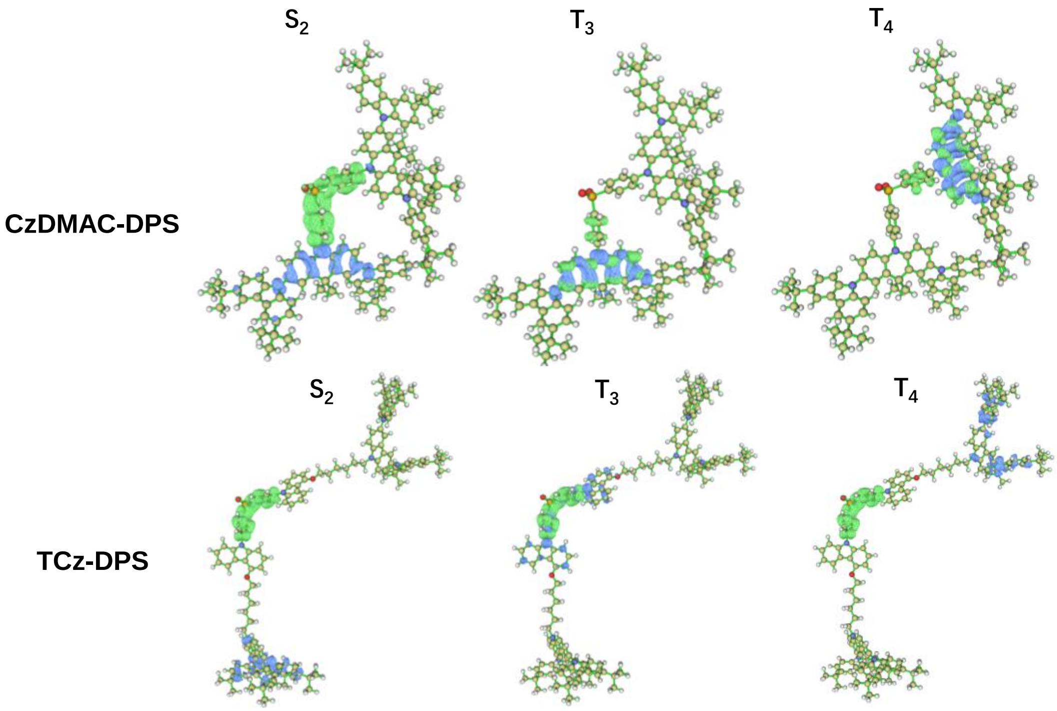

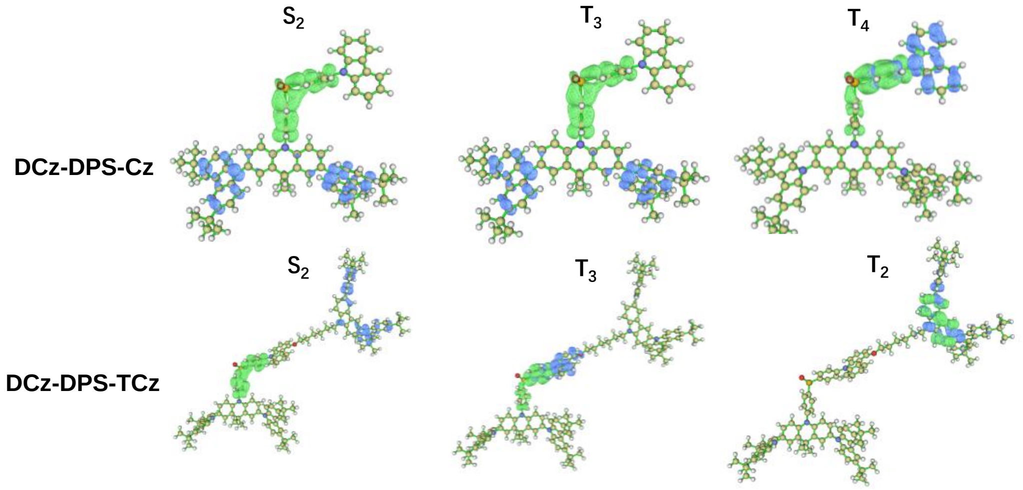

Fig. S22 NTO analysis of the studied four dendronized emitters.

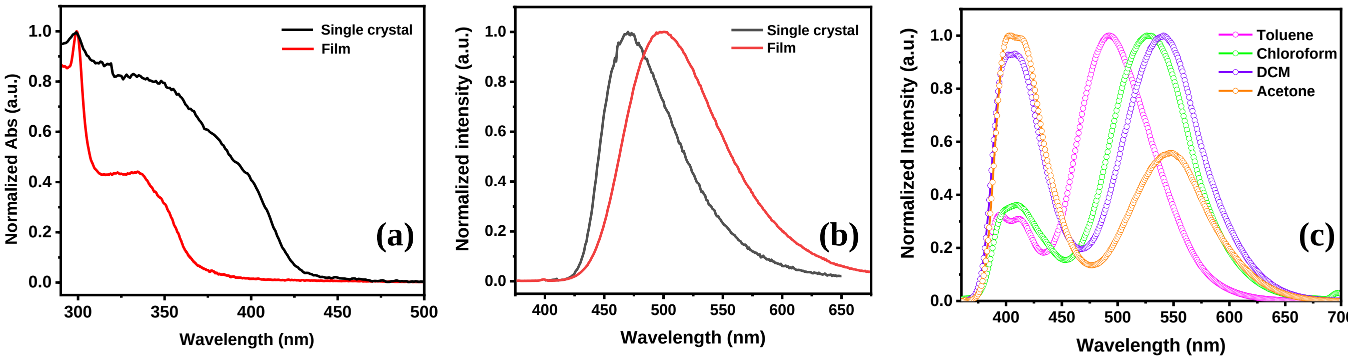

Fig. S23 UV-vis absorption (a) and photoluminescence spectra (b) of **DCz-DPS-TCz** in film and single crystal. (c) Photoluminescence spectra in toluene, chloroform, acetone and dichloromethane (DCM) solution of **DCz-DPS-TCz**.

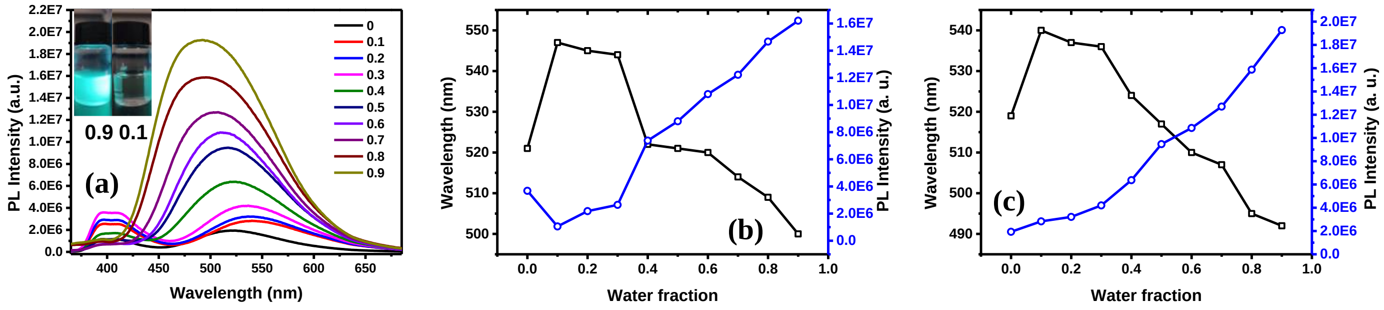

Fig. S24 (a) PL spectra of **DCz-DPS-TCz** in water/THF mixtures with different fractions of water; the inset is fluorescent images of 0.9 and 0.1 water/THF solution mixtures under UV light irradiation. (b, c) **DCz-DPS-Cz** and **DCz-DPS-TCz** statistics of PL intensity and emission peak.

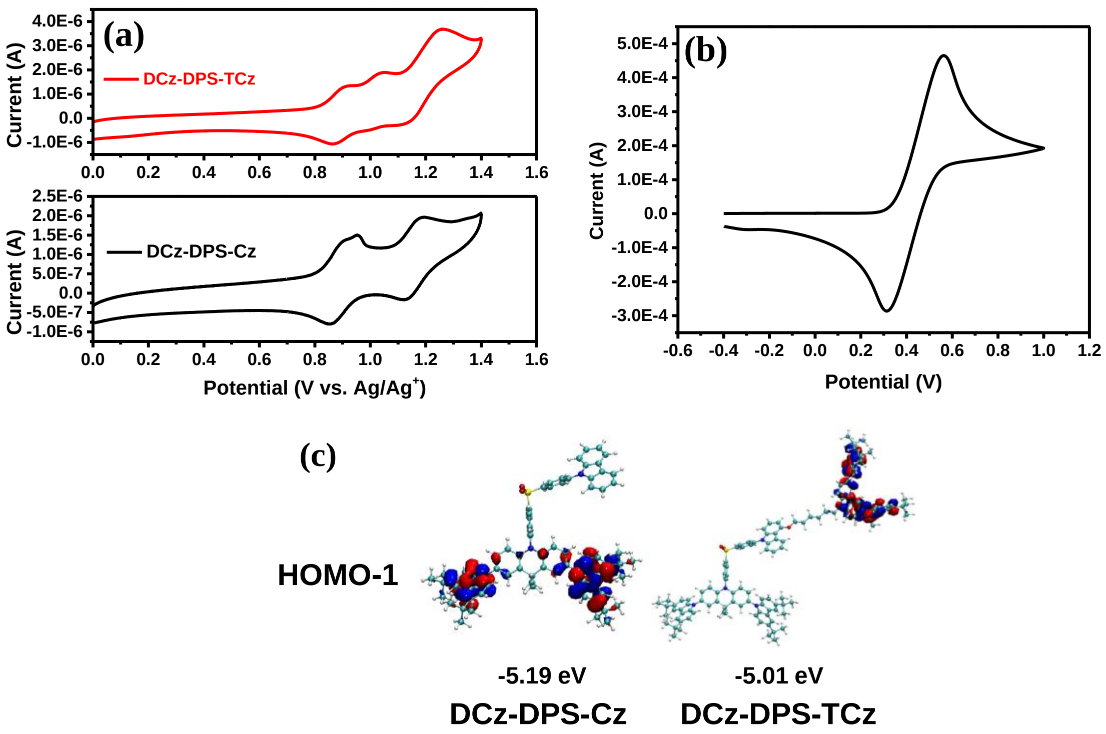

Fig. S25 (a) Cyclic voltammograms of the two dendronized emitters in degassed anhydrous acetonitrile; (b) the Fc^{+}/Fc external standard in acetonitrile; (c) the HOMO-1 orbitals of **DCz-DPS-Cz** and **DCz-DPS-TCz**.

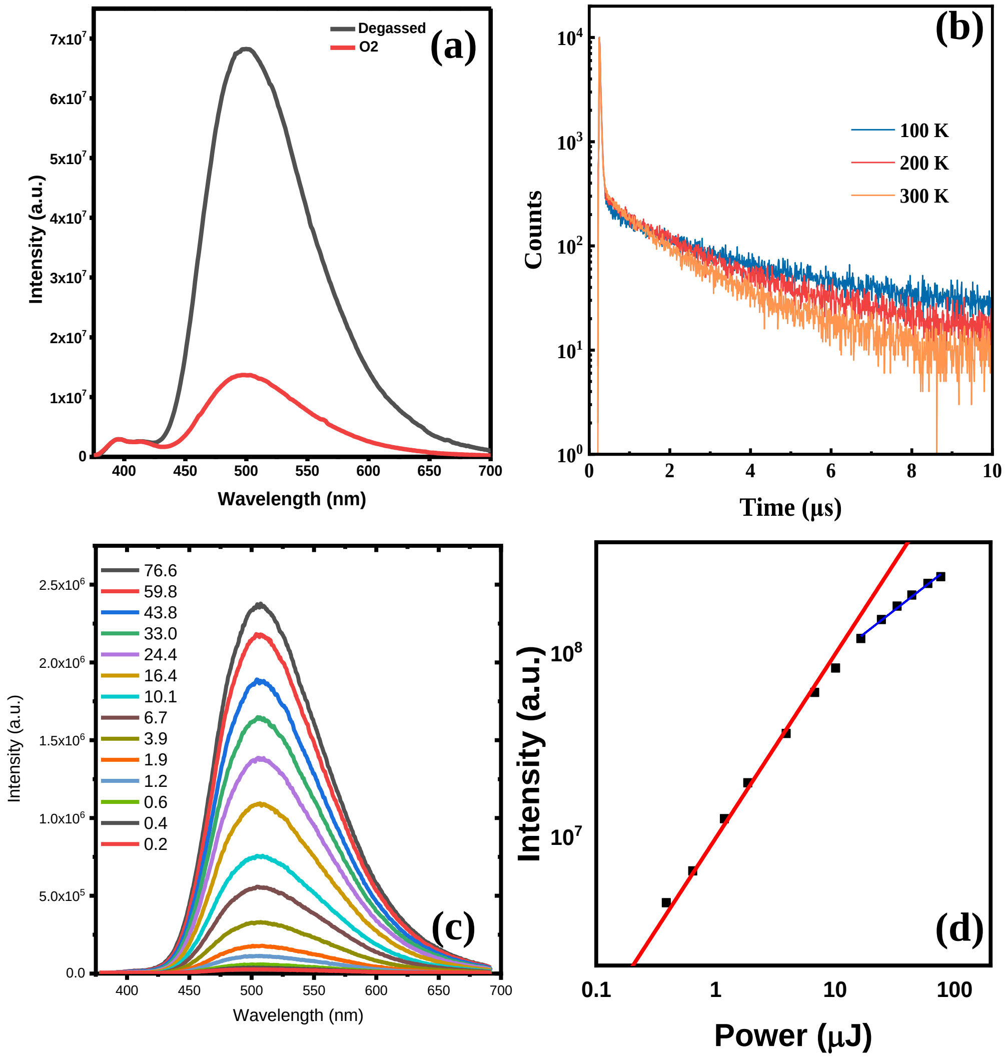

Fig. S26 (a) Photoluminescence spectra in degassed and oxygenated toluene solution of **DCz-DPS-TCz**. (b) Photoluminescence decays in neat films of **DCz-DPS-TCz**. (c, d) PL spectra and dependence of DF intensity with excitation power (0.2 – 76.6 μJ) of **DCz-DPS-TCz** in neat films.

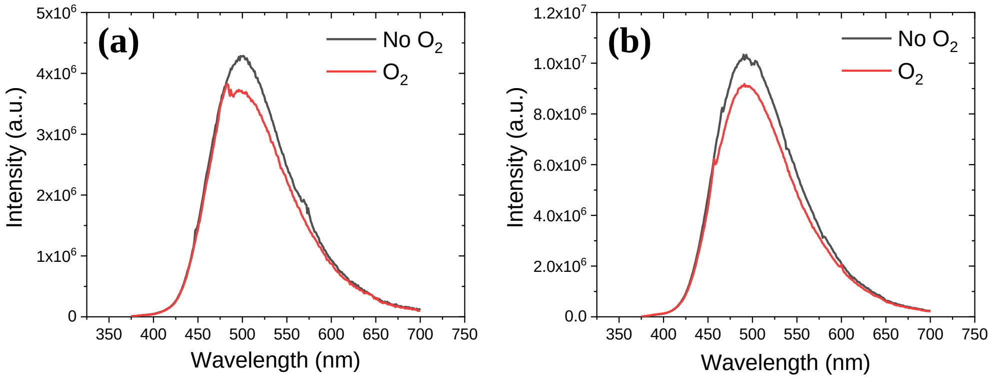

Fig. S27 Steady state emission oxygen dependence of (a) **DCz-DPS-Cz** and (b) **DCz-DPS-TCz** in neat film.

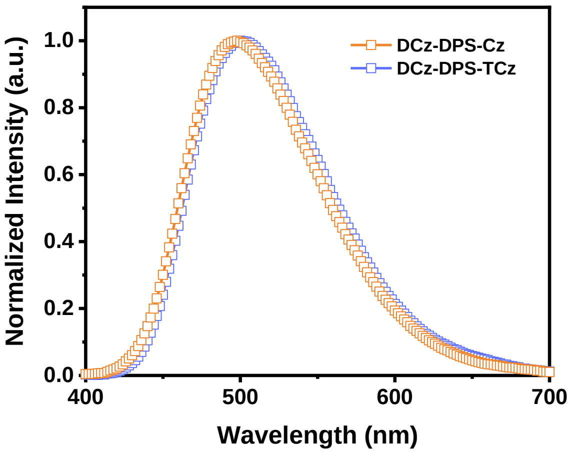

Fig. S28 Normalized EL spectra of **DCz-DPS-Cz** and **DCz-DPS-TCz** at 5 V.

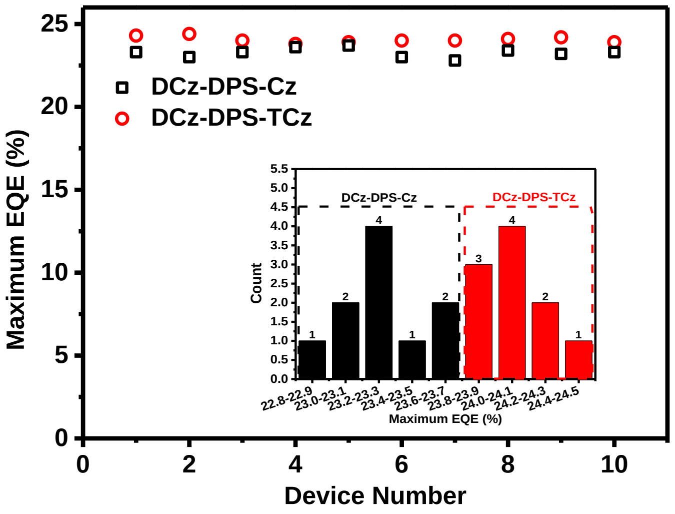

Fig. S29 Maximum EQEs for solution-processed OLEDs using **DCz-DPS-Cz** and **DCz-DPS-TCz** as emitter measured from 10 devices. The average maximum EQE values are 23.3% and 24.0% with distribution from 22.8 to 23.7% and 23.8% to 24.5%, corresponding to variations of ±0.45% and ±0.35%, respectively.

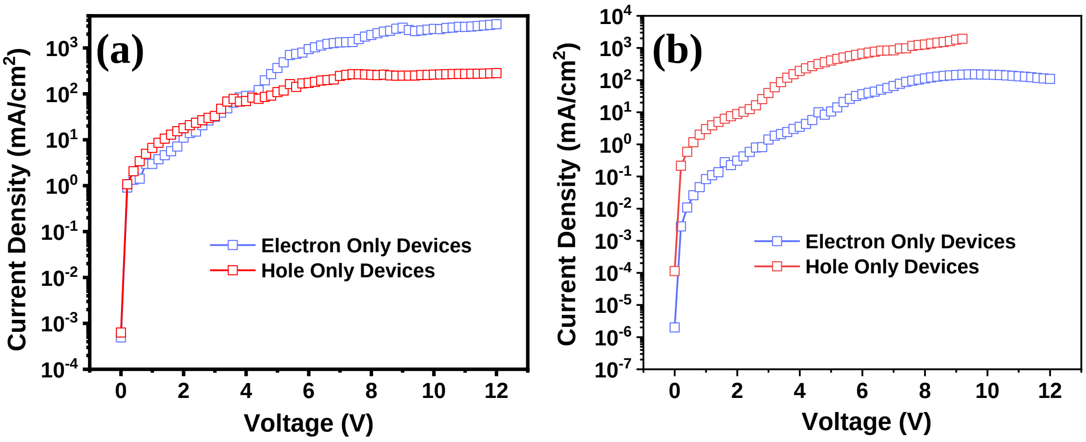

Fig. S30 *J-V* curves of the electron- and hole-only devices for **DCz-DPS-Cz** (a) and **DCz-DPS-TCz** (b). Electron-only devices: ITO/ZnO (20 nm)/neat film (40 nm)/LiF (0.9 nm)/Al (120 nm). Hole-only devices: ITO/PEDOT:PSS (40 nm)/neat film (40 nm)/MoO_3 (2.0 nm)/Al (120 nm).

References:

[1] P. Pander, P. Data, F. B. Dias, *J. Vis. Exp*. **2018**, *142*, e56614.

[2] M. J. Frisch, G. W. Trucks, H. B. Schlegel, G. E. Scuseria, M. A. Robb, J. R. Cheeseman, G. Scalmani, V. Barone, B. Mennucci, G. A. Petersson, H. Nakatsuji, M. Caricato, X. Li, H. P. Hratchian, A. F. Izmaylov, J. Bloino, G. Zheng, J. L. Sonnenberg, M. Hada, M. Ehara, K. Toyota, R. Fukuda, J. Hasegawa, M. Ishida, T. Nakajima, Y. Honda, O. Kitao, H. Nakai, T. Vreven, J. A. Jr. Montgomery, J. E. Peralta, F. Ogliaro, M. Bearpark, J. J. Heyd, J. J. Brothers, K. N. Kudin, V. N. Staroverov, T. Keith, R. Kobayashi, J. Normand, K. Raghavachari, A. Rendell, J. C. Burant, S. S. Iyengar, J. Tomasi, M. Cossi, N. Rega, J. M. Millam, M. Klene, J. E. Knox, J. B. Cross, V. Bakken, C. Adamo, J. Jaramillo, R. Gomperts, R. E. Stratmann, O. Yazyev, A. J. Austin, R. Cammi, C. Pomelli, J. W. Ochterski, R. L. Martin, K. Morokuma, V. G. Zakrzewski, G. A. Voth, P. Salvador, J. J. Dannenberg, S. Dapprich, A. D. Daniels, O. Farkas, J. B. Foresman, J. V. Ortiz, J. Cioslowski, D. J. Fox, Gaussian, Inc., Wallingford CT, 2010.

[3] J. Chen, H Tian, Z. Yang, J. Zhao, Z. Yang, Y. Zhang, M. P. Aldred, Z. Chi, *Adv. Opt. Mater.* **2020**, *9* (2), 2001550.

[4] K. Albrecht, K. Matsuoka, D. Yokoyama, Y. Sakai, A. Nakayama, K. Fujita, K. Yamamoto, *Chem. Commun.* **2017**, *53*, 2439-2442.
